# Endothelial Cells in Emerging Viral Infections

**DOI:** 10.3389/fcvm.2021.619690

**Published:** 2021-02-24

**Authors:** Johanna Hol Fosse, Guttorm Haraldsen, Knut Falk, Reidunn Edelmann

**Affiliations:** ^1^Norwegian Veterinary Institute, Oslo, Norway; ^2^Department of Pathology, Oslo University Hospital, Oslo, Norway; ^3^Department of Pathology, University of Oslo, Oslo, Norway; ^4^AquaMed Consulting AS, Oslo, Norway; ^5^Department of Clinical Medicine, Centre for Cancer Biomarkers CCBIO, University of Bergen, Bergen, Norway

**Keywords:** endothelium, virus, SARS-CoV-2, inflammation, vascular dysfunction and damage, emerging infections

## Abstract

There are several reasons to consider the role of endothelial cells in COVID-19 and other emerging viral infections. First, severe cases of COVID-19 show a common breakdown of central vascular functions. Second, SARS-CoV-2 replicates in endothelial cells. Third, prior deterioration of vascular function exacerbates disease, as the most common comorbidities of COVID-19 (obesity, hypertension, and diabetes) are all associated with endothelial dysfunction. Importantly, SARS-CoV-2's ability to infect endothelium is shared by many emerging viruses, including henipaviruses, hantavirus, and highly pathogenic avian influenza virus, all specifically targeting endothelial cells. The ability to infect endothelium appears to support generalised dissemination of infection and facilitate the access to certain tissues. The disturbed vascular function observed in severe COVID-19 is also a prominent feature of many other life-threatening viral diseases, underscoring the need to understand how viruses modulate endothelial function. We here review the role of vascular endothelial cells in emerging viral infections, starting with a summary of endothelial cells as key mediators and regulators of vascular and immune responses in health and infection. Next, we discuss endotheliotropism as a possible virulence factor and detail features that regulate viruses' ability to attach to and enter endothelial cells. We move on to review how endothelial cells detect invading viruses and respond to infection, with particular focus on pathways that may influence vascular function and the host immune system. Finally, we discuss how endothelial cell function can be dysregulated in viral disease, either by viral components or as bystander victims of overshooting or detrimental inflammatory and immune responses. Many aspects of how viruses interact with the endothelium remain poorly understood. Considering the diversity of such mechanisms among different emerging viruses allows us to highlight common features that may be of general validity and point out important challenges.

## Introduction

As the human population expands, a closer proximity to wild animals and their habitat has resulted in a more frequent spillover of viral infections to humans and livestock. At the same time, our mobility has increased. This augments the risk that emerging infections can spread globally. As a result, evolving infections pose a significant threat to human and animal health and the global economy. This is clearly illustrated by the ongoing severe acute respiratory syndrome coronavirus 2 (SARS-CoV-2) pandemic (>2.1 million deaths). Other recent epidemics include the 2014-2016 Ebola outbreak in West Africa (>11,000 deaths), the MERS-CoV outbreak in Saudi Arabia 2012-2019 (>850 deaths), and regular outbreaks of highly fatal Nipah virus infection in Bangladesh and surrounding countries, among others (1).

A characteristic feature of SARS-CoV-2 is that the virus, in addition to infecting airway epithelial cells, also infects endothelial cells (2–4). This infection pattern can be recapitulated in the laboratory, as SARS-CoV-2 infects and replicates in human capillary organoids (5). However, it is not known if replication in endothelial cells is a ubiquitous feature of disease or limited to certain patient groups or clinical presentations. Coronavirus disease 2019 (COVID-19) has an increased case fatality rate in individuals suffering from conditions associated with endothelial dysfunction (diabetes, hypertension, and cardiovascular disease) (6). Furthermore, typical clinical signs in severe COVID-19 are compatible with breakdown of vascular function (multi-organ endothelial damage, thrombosis and angiopathies, dysregulated inflammation, and pulmonary oedema) (2, 3, 7). To this end, it is highly relevant to address the role of the vascular compartment in COVID-19 (7, 8).

SARS-CoV-2's ability to infect endothelial cells is shared by many emerging viruses with great relevance to human and animal health. For example, henipavirus infection leads to widespread infection of endothelial and vascular smooth muscle cells, with extensive vasculitis and occasional endothelial syncytia formation (9, 10). In hantavirus pulmonary syndrome, there is severe dysregulation of vascular permeability and strong expression of viral antigens in the pulmonary microvasculature (11). Substantial endothelial expression of viral antigens is also observed in several emerging infections of animals, which have a major impact on animal welfare and sustainable food production. Highly pathogenic avian influenza virus (HPAIV, also relevant because of its zoonotic potential) (12) and pathogenic variants of infectious salmon anaemia virus (ISAV) (13) are prime examples of viruses that infect endothelial cells of production animals. Other viruses have a more generalised cell tropism, but show some degree of endothelial infection during natural infection. Relevant examples include the flaviviruses (such as dengue virus) (14) and filoviruses (Ebola and Marburg viruses) (15). Table 1 provides a list of viral pathogens discussed in the current article and associated pathological findings of vascular disease. However, the complete list of all viruses that target the endothelium would be much more extensive.

**Table 1:** Viruses that infect endothelial cells and related vascular pathological findings.

**Virus (Family)**	**EC infection**	**Vascular pathological features**	**References**
SARS-CoV-2 (coronaviridae)	One of several cell types infected	Intra-endothelial virus particles in all examined tissues; Multi-organ vascular involvement; Endotheliitis with perivascular T cell and mononuclear cell infiltrates; Severe endothelial injury with disrupted cell membranes, and apoptotic bodies; Widespread thrombosis, congestion, and microangiopathy; Dysregulated inflammation	(2–4)
Nipah and Hendra henipavirus (paramyxoviridae)	Major cell type infected	Widespread expression of viral antigens in endothelial and vascular smooth muscle cells; Multi-organ vascular involvement; Endotheliitis affecting small arteries, arterioles, capillaries, and venules; Focal neutrophil and mononuclear infiltrates; Severe endothelial injury with segmented destruction, mural necrosis, and fragmented nuclei; Necrosis and haemorrhages adjacent to damaged endothelium; Endothelial syncytia, multinucleated giant cells	(9, 10)
Hantavirus (hantaviridae)	Major cell type infected	Viral antigens, viral inclusions bodies, and virus particles in pulmonary capillary and small vessel endothelium; Less frequent presence of endothelial viral antigens in extra-pulmonary tissues, with kidney being the most affected location; Pulmonary and generalised vascular congestion, but histopathological changes confined to lungs; Alveolar and interstitial oedema; Mononuclear cell infiltrates without visible endotheliitis; Occasional swollen or enlarged endothelial cells, but no evidence of necrosis or microvascular thrombosis	(11)
Influenza A, H5N1, H7N1 (orthomyxoviridae)	Major cell type infected	Viral antigens in blood and lymphatic endothelial cells of all examined tissues; Viral particles budding from the luminal side; Microvascular thrombosis; Multi-organ vascular involvement with widespread necrotic and haemorrhagic foci and oedema; Inflammation, except in per-acute disease	
Infectious salmon anaemia virus (orthomyxoviridae)	Major cell type infected	Viral antigens and virus particles in endothelial cells of the primary and secondary vascular system; Multi-organ vascular involvement, including bleeding, congestion, and oedema; Severe anaemia; Absence of perivascular leukocyte infiltrates	
Ebolavirus (filoviridae)	One of many cell types infected	Endothelial infection becomes evident after onset of symptoms; Intra-endothelial viral inclusion bodies and virus particles; Absence of inflammation and morphological evidence of EC damage; Petechiae and ecchymoses on skin and mucous membranes, internal haemorrhages, fibrin deposition suggestive of consumptive coagulopathy; Multi-organ necrotic foci	(15, 16)
Dengue virus (flaviviridae)	One of many cell types infected	Viral antigens in endothelial cells of lung, liver, and heart; Diffuse haemorrhage, oedema, and focal necrosis; Mononuclear cell infiltrates	(14, 17)
Zikavirus, West Nile virus (flaviviridae)	Limited evidence of *in vivo* endothelial infection		

In addition, severe viral disease more often than not includes signs related to dysregulated vascular function. Typical presentations include dysregulated blood flow, uncontrolled inflammation and vascular permeability, and microvascular thrombosis and bleeding. Importantly, vascular function may also be disturbed in the absence of endothelial infection. This can result from signalling from infected cells or collateral damage when immune cells attempt to limit infection. In conclusion, the question of how viruses target the endothelium, either directly or as bystanders, appears central to understand the pathogenesis of disseminated viral disease.

Complementary to several recent reviews that discuss the vascular component of COVID-19 [e.g., (8, 18, 19)], we here broaden our perspective to consider the diversity by which emerging viruses interact with endothelial cells. Briefly, we review features of endothelial cells relevant to their role in the pathogenesis of viral disease, summarise what is known about viral properties that regulate their ability to infect endothelial cells, and discuss how such tropism may affect the course of infection and disease development. Our aim is not to provide a complete overview of all viral interactions with endothelium, but rather to provide examples that illustrate the range of such interactions, highlight general principles, and point to unanswered questions related to disease development.

## Endothelial Function in Homeostasis and Inflammation

### Endothelial Cells Are Gatekeepers of the Blood-Tissue Barrier

Endothelial cells line the inner surface of all blood and lymphatic vessels. This single cell layer, the endothelium, creates a semi-permeable barrier between blood or lymph and its surrounding tissues. In an adult human, the vasculature contains ~6 x 10^11^ endothelial cells (20), covering a surface area of 4,000–7,000 m^2^. The vascular network is a highly branched closed circulation that extends into all organs, nourishes every tissue, and provides a gateway for extravasation of fluids, solutes, macromolecules, hormones, and immune cells. Consequently, all possible entry ports of viral infection are in close contact with endothelial cells. A good example is the lower respiratory tract, where intimate apposition between alveolar epithelial and lung microvascular endothelial cells is required for efficient alveolar gas exchange. Microvascular beds, composed of arterioles, capillaries, and post-capillary venules, make up the greatest surface of the vascular circulation by far and is where most physiological processes brought on by the endothelium occur (21). This is also where most virus-induced vascular pathology manifests.

### Endothelial Profiles Vary Between Vascular Beds, Branches, and Activation States

Endothelial cells show tissue- and vascular bed-specific profiles that are likely to regulate their susceptibility and permissibility to viral infection (22). For example, the observation of dengue antigens in liver sinusoids (14) during natural infection should be considered in light of the dengue virus' ability to exploit scavenger receptors for internalisation (23, 24). The vast diversity among endothelial cells arises through a combination of inputs that impose phenotypes of variable durability (25). Some phenotypes are mitotically stable, like the epigenetic modifications that define arterial and venous cells (26, 27). Others provide memories of past stimulations, like biologically active proteins with long half-lives stored in Weibel-Palade bodies (28). Finally, endothelial phenotypes may also be highly dynamic, like tip-stalk cell phenotypes in angiogenic sprouting (29). Similar to many aspects of endothelial heterogeneity, the ability to regulate critical vascular functions is dynamic and arises from cues within the extracellular environment (28, 30). Thus, maintaining blood fluidity, regulating blood flow, controlling trans-endothelial extravasation of plasma proteins, and controlling leukocyte trafficking all respond to signals generated during homeostasis, hypoxia, inflammation, and repair. Importantly, endothelial cells may reside in a resting phenotype over long periods of time and still maintain the ability to orchestrate dramatic responses when activated (31).

### Endothelial Cells Are Central Regulators of Vascular Function

**Figure 1:**
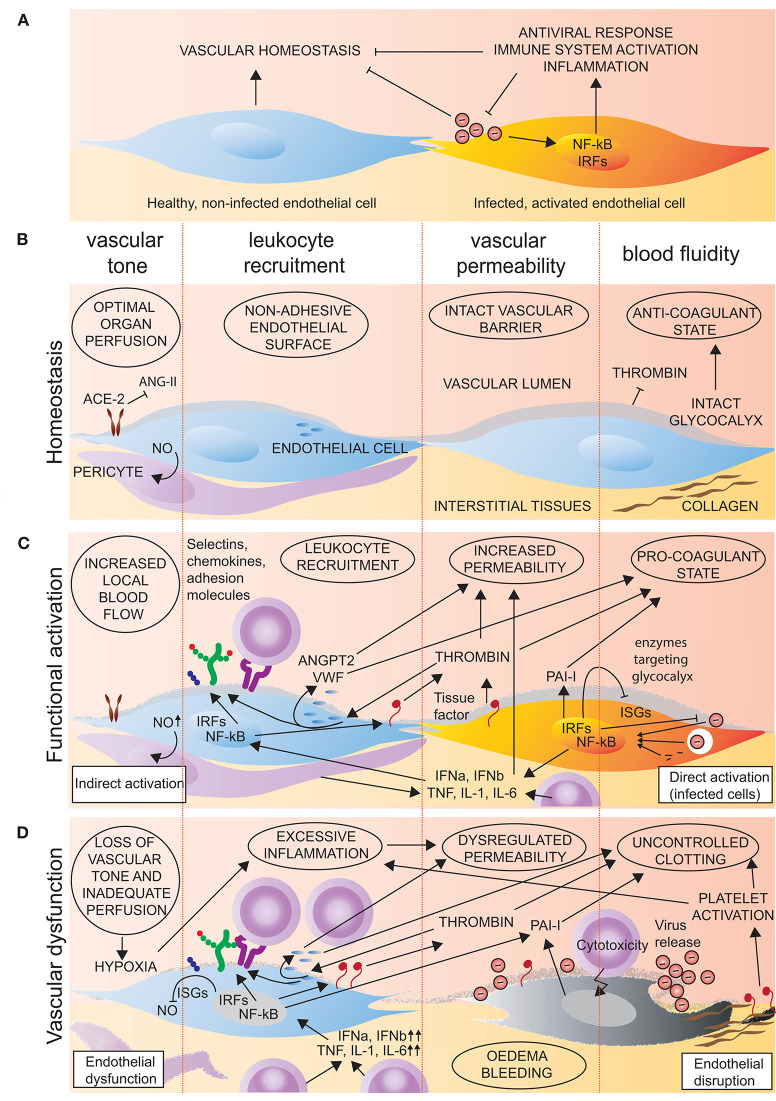
Endothelial function in homeostasis and viral infection. **(A,B)** In homeostasis, endothelial cells promote a non-adherent vascular surface that promotes tissue perfusion, limits inflammation and the transvascular movement of cells and proteins, and prevents clotting. **(A,C)** Upon viral infection or tissue damage, endothelial cells are activated directly by viral components or indirectly by soluble mediators (IL-1, TNF, IL-6, IFNa, IFNb). Such activation initiates signalling that culminates in activation and nuclear translocation of IRF and NF-kB transcription factors. This results in expression of interferon-stimulated genes (ISGs) and inflammatory mediators (e.g., tissue factor, selectins, chemokines, and adhesion molecules) that promote antiviral responses, clotting, vascular permeability, and leukocyte recruitment. In addition, type I activation of endothelial cells, for example by thrombin, causes a rapid release of pre-synthesised molecules (e.g., VWF, ANGPT2, P-selectin, and CXCL8) with similar effects. **(A,D)** In the case of overwhelming or persistent activation, endothelial cells may become dysfunctional, resulting in loss of control of vascular tone, permeability, and coagulation. In addition, direct viral damage to the endothelium further disrupts the vascular barrier and exposes pro-thrombotic factors (tissue factor, collagen) that exacerbate dysfunctional coagulation.

Endothelial cells control vascular functions, and tissue damage or infection stimulates an endothelial phenotype that supports the protective host response. However, overwhelming or persistant activation can lead to endothelial dysfunction and contribute to pathology (Figure 1).

#### Regulation of Blood Flow

Arterial endothelium plays an essential role in regulating blood flow by controlling perivascular smooth muscle cell contraction. The specific blood-flow mediators released by endothelial cells vary in time and space, but the principal vaso-relaxant is nitric oxide (NO) (32). NO is also linked to various other endothelial functions, and reduced NO bioavailability is a central feature of endothelial dysfunction (33). Indeed, a non-invasive test for measuring the NO-mediated response to alterations in blood pressure (brachial artery flow-mediated dilation, FMD) is commonly used to define endothelial dysfunction (34). In a recent position paper by the European Society for Cardiology, FMD was proposed as a potential tool for risk stratification in COVID-19 (7). The renin-angiotensin system is also a key player in the regulation of blood pressure and organ perfusion. Excessive activation of the renin-angiotensin system by angiotensin II (ANG-II) causes vasoconstriction, as well as vascular and cardiac damage. However, its counterregulation by the angiotensin converting enzyme 2 (ACE2), the surface protein used by SARS-CoV-2 for cellular attachment (35–37), and its products, angiotensin 1-7 and angiotensin 1-9, balances signalling to promote the perfusion of central organs like heart and kidney (38).

#### Maintenance of Blood Fluidity

Under homeostatic conditions, the endothelium actively inhibits coagulation (32). Because microvascular thrombosis is a clinical feature of many viral infections and central to COVID-19 (39), we here provide a brief overview of the mechanisms involved. First, endothelial cells bind and present tissue factor pathway inhibitors that block the action of the factor-VIIa-tissue-factor complex (32). Second, endothelial cells produce and display heparan sulphate proteoglycans in the glycocalyx that bind anti-thrombin III, enabling inhibition of thrombin molecules generated by the coagulation cascade (32). Third, endothelial cells synthesise and present thrombomodulin, a cell surface-bound protein that prevents thrombin from cleaving fibrinogen, a key step in blood clot formation. Furthermore, this thrombomodulin-bound thrombin efficiently activates protein C, generating an enzyme that destroys certain clotting factors, also inhibiting coagulation (32). Fourth, thrombin inactivation by endothelial cells prevents platelet activation, another critical element in maintaining blood fluidity. Endothelial cells also produce and sequester von Willebrand factor (VWF) in Weibel-palade bodies. VWF is a large multimeric glycoprotein that plays a central role in coagulation, among others by strengthening the binding between platelets and collagen in the basement membrane (32). Finally, endothelial-produced NO, prostacyclin, and prostaglandin E2 synergistically inhibit platelet adhesion and aggregation (32). Importantly, in response to pro-inflammatory activation, endothelial cells change from an anti-coagulant to a pro-coagulant phenotype (Figure 1C). Increased tissue factor expression and exposure of extracellular matrix components like collagen initiate the extrinsic coagulation cascade activation and increased production of thrombin and fibrin. The resulting pro-thrombotic state is further exaggerated by an elevation of plasminogen activator inhibitor 1 (PAI-I) that suppresses the fibrinolytic system (40). Finally, secretion of VWF promotes platelet activation and additional propagation of the coagulation cascade.

#### Control of Vascular Permeability

Microvascular endothelial cells also regulate the extravasation of fluids, solutes, and plasma proteins. Accordingly, dysregulation of microvascular function mediates oedema and bleeding in viral disease (41). The maintenance of microvascular integrity relies on crosstalk between two key components of the endothelium, the glycocalyx and the intercellular junctions. First, the luminal surface of all vascular endothelial cells is covered by the glycocalyx. This is a thick hydrated layer of glycoproteins with acidic oligosaccharides and terminal sialic acids that confer a net negative charge (42). Of note, these highly abundant sialic acids are frequently exploited by viruses to attach to the cellular surface (43). The glycocalyx also contains membrane-bound proteoglycans with associated glycosaminoglycans like heparan sulphates (42). It forms a gel-like interface between the plasma membrane and the blood that is central to maintaining proper barrier function, and its disruption leads to vascular leakage (42, 44). Second, the conformation of endothelial intercellular junctions is highly dynamic and responds to a range of stimuli (45). Tight junction molecules regulate paracellular permeability to ions and small molecules, and are therefore most prominent at sites where strict regulation of vascular permeability is essential, like the blood-brain barrier (46). In contrast, adherens junction molecules are evenly distributed along the vascular tree, and mediate permeability to large molecular weight plasma components (46). Adherens junctions are also hubs for the integration of cell-cell adhesion, cytoskeletal reorganisation, and intracellular signalling (45). The highly endothelial cell-specific vascular endothelial (VE)-cadherin (47) is a central component of adherens junctions and has a crucial role in regulating the permeability to plasma proteins across intact endothelium (48).

#### Orchestration of Leukocyte Recruitment

Venular endothelial cells keep tight control of leukocyte trafficking from blood to tissues and orchestrate local inflammatory responses, for example in viral pneumonia. For leukocytes to cross the vessel wall, they must first adhere to the endothelial surface. This process takes place in post-capillary venules of inflamed tissues and has been entitled the multistep adhesion cascade (49). Briefly, the capture and rolling of leukocytes on endothelial surfaces is mediated by P-, E-, and L-selectins, which bind glycosylated ligands. This happens in a dynamic process, forming and breaking bonds that support capture and rolling under shear stress (50). P- and E-selectin appear to be the most important selectins expressed on activated post-capillary venules in non-lymphoid tissues (51). Rolling leukocytes gets into close contact with endothelial cells, and this proximity allows inside-out activation of leukocyte integrins by glycocalyx-presented chemokines. In addition to producing chemokines themselves, endothelial cells are also able to capture chemokines produced by stromal cells, transcytose them, and present them on their luminal surface (52). This transcytosis of chemokines communicates the nature of extravascular inflammatory processes to circulating leukocytes and is mediates by atypical chemokine receptors. A key example is the Duffy antigen receptor for chemokines that binds both CC and CXC inflammatory chemokines and is required for optimal chemokine-induced leukocyte transmigration (52). Cytokines produced by resident leukocytes also stimulate endothelium to sequentially upregulate adhesion molecules. These adhesion molecules bind chemokine-activated leukocyte integrins and mediate firm adhesion and arrest.

#### Endothelial Activation May Be Rapid or Sustained

Endothelial responses to inflammatory stimuli can be classified as type I (in response to e.g., thrombin) or type II (in response to inflammatory cytokines). Type I responses rely on the release of pre-synthesised molecules and do not depend on new gene expression. This very rapid activation allows recruitment of leukocytes within minutes of the initiating stimulus (53). Type I responses are typically mediated by G protein-coupled receptors, whose transient signalling ensures the short-lived nature of the response (32). G-protein coupled signalling results in elevation of cytosolic free Ca^2+^. Free Ca^2+^ interacts with calmodulin to drive the phosphorylation of myosin light chain that initiates the exocytosis of the predominant endothelial storage granule, the Weibel-Palade body. In addition to the pro-thrombotic molecule VWF, a subset of Weibel-Palade bodies also contains angiopoietin 2 (ANGPT2) (54), P-selectin (55), and the powerful neutrophil chemoattractant CXCL8 (56) that together drive vascular destabilisation and rapid leukocyte recruitment. The rise in intracellular Ca^2+^ also leads to NO and prostacyclin production, thus promoting increased blood flow and vascular leakage. In contrast, type II responses constitute the classical response to pro-inflammatory cytokines, like interleukin (IL)-1 and tumour necrosis factor (TNF), resulting in activation of the inflammatory transcription factors NF-kB and AP1 and *de novo* gene expression. Such activation gives rise to a more sustained and efficient inflammatory response, but with the same consequences of increased blood flow, vascular leakage, and a coordinated leukocyte recruitment (Figure 1C). Moreover, both type I and type II activation of endothelial cells increase vascular permeability, at least in part by stimulating the formation of fibrillar adhesions to the extracellular matrix that destabilise endothelial adherens junctions and allow extravasation of plasma proteins (57).

#### Endothelial Signalling Pathways That Regulate Normal Vascular Function

In addition to signalling induced by inflammatory cytokines, a number of signalling pathways integrate to regulate endothelial function, including angiopoietin-1 (ANGPT1)/TIE2- (58), vascular endothelial growth factor (VEGF)- (59), WNT/β-catenin-, sphingosine-1-phosphate (S1P)-, and platelet-derived growth factor (PDGF)-signalling (60). In homeostasis, such highly interlinked signalling promotes stable endothelial junctions, as well as interactions with neighbouring mural cells. Endothelial junctional stability primarily relies on VE-cadherin homophilic interactions (45, 46). In a resting vessel, VE-cadherin also directly interacts with VEGFR2, reducing its signalling potential and protecting the cell from the destabilising effect of VEGF-A (61). Interestingly, in this setting, VEGF-A mediates anti-apoptotic signalling through PI3K/AKT-induced eNOS phosphorylation; exemplifying the complexity of the signalling involved in endothelial cell maintenance (62). VEGFR2 and VE-cadherin also merge in a mechano-sensory complex together with platelet EC adhesion molecule 1 (also known as CD31) that mediates the endothelial response to shear stress (63). Moreover, ANGPT1/TIE2-, S1P-, and PDGF-signalling enhance pericyte recruitment and integrin-mediated adhesion to the extracellular matrix, further stabilising vessel integrity (64–67). Finally, endothelial cells also express connexins that form intercellular gap junctions and contribute to the close communication between adjacent endothelial cells, pericytes, and vascular smooth muscle cells (68, 69).

### Endothelial Dysfunction Is a Central Component of Many Viral Syndromes

A common feature of viruses that infect endothelial cells is their ability to cause severe multi-organ disease. The clinical features of end-stage viral disease are often similar, with hypoperfusion, oedema, bleeding, and thrombosis, all indicating breakdown of central vascular functions (Figure 1D). Typical vascular-associated histopathological features of different viral infections are summarised in Table 1. This section reviews common viral syndromes, with emphasis on their vascular component.

#### Acute Respiratory Distress Syndrome

In all virus pandemics of the last two decades (Influenza A H1N1; SARS-CoV, MERS-CoV, and SARS-CoV-2) a high proportion of affected patients have developed severe illness and acute respiratory distress syndrome (ARDS) (70–72). ARDS is a complication to viral pneumonia, characterised by pulmonary oedema, hypoxaemia, and increasing respiratory failure, resulting in progressive multi-organ failure and often fatal outcomes [exceeding 50% (73)]. Breakdown of the pulmonary microvascular barrier function is a central feature of ARDS (74, 75). The histopathological correlates are disruption of the alveolar epithelial and vascular barriers and accumulation of protein-rich exudate within the interstitium and alveolus (76). This eventually leads to diffuse alveolar damage (77, 78), which is the pathologic hallmark of ARDS. Respiratory viruses initially infect the upper airway epithelium, but the importance of this step to alveolar damage is debated. Instead, it might be that the epithelium merely functions as a portal of entry, while the alveolar damage primarily results from compromised vascular integrity (79). The release of potent pro-inflammatory cytokines and chemokines by resident macrophages, epithelial, and endothelial cells leads to the recruitment of both innate and adaptive immune cells (80). These cells cause further exacerbation of tissue damage. Specific cytokine profiles vary by the virus, but a general phenomenon for all recent pandemic viruses is their ability to induce an excessive early cytokine response (81, 82). Together with immune cell recruitment, this excessive response strongly correlates with poor outcome. In H1N1 infection, pulmonary endothelial cells play a central role in regulating both innate immune cell recruitment as well as innate cytokine and chemokine production (83). Hence, it is tempting to speculate if this also holds true for coronavirus-induced ARDS. A study comparing post mortem-collected tissues from H1N1 and COVID-19 patients found more severe disruption of endothelial morphology in COVID-19 patients (3), supporting this hypothesis. Clinical features of the hantavirus pulmonary syndrome very much mimic those of influenza- and coronavirus induced ARDS (11). Nevertheless, it does not appear to be mediated by release of inflammatory cytokines or morphological disruption of the endothelium (11, 41). Instead, the dramatic disruption of vascular barrier function is mediated by direct viral manipulation of inter-cellular junctions (41, 84), discussed in more detail in section 4.3.1.

#### Microvascular Thrombosis and Endotheliitis

Disseminated intravascular coagulation and thrombotic microvascular occlusion are complications of severe systemic inflammation and a sequela to most infections discussed in this article (Table 1). Notably, COVID-19 has been strongly associated with thrombosis (85), more so than other coronaviruses and H1N1 (39). This is not entirely surprising, as severely ill SARS-CoV-2-infected patients have intra-endothelial viral inclusion bodies, endothelial apoptosis, and inflammation (endotheliitis) (2–4). The extensive morphological vascular changes affect a range of organs, from the lung to the gastrointestinal tract, suggesting widespread endothelial activation and damage. The true prevalence of microthrombosis in COVID-19 is still unknown, as most studies to date do not include systematic and comprehensive investigation protocols (39). However, multiple reports have shown cumulative incidences of thrombotic events around 50% (86), even when thrombo-profylaxis has been administered. In fact, in one study 56% of patients receiving full anticoagulation were diagnosed with pulmonary embolism (87). Interestingly, these rates are similar to those reported in patients with severe sepsis and shock (39, 88).

#### Systemic Inflammatory Response Syndrome and Multi-Organ Failure

As the disruption of vascular functions becomes more severe, many viral infections culminate in a typical systemic inflammatory response syndrome, resembling severe sepsis. In COVID-19, it soon became evident that patients with severe disease fulfilled all diagnostic criteria for sepsis and septic shock, even in the absence of bacterial co-infections (89). This systemic inflammatory response syndrome reflects overwhelming activation by inflammatory cytokines and other activators. Initial peripheral vasoconstriction is followed by uncontrolled vascular dilation, functional hypovolaemia, and circulatory failure, eventually culminating in multi-organ failure and death. Nevertheless, cytokine levels in critically ill COVID-19 patients were lower than in patients with bacterial sepsis (90). Therefore, it is possible that the severe direct viral damage to the endothelium (3) further adds to the cytokine-stimulated breakdown of vascular function in COVID-19. This parallel between bacterial and viral sepsis is maintained in dengue vascular permeability syndrome. This syndrome is characterised by severe hypovolemic shock, in addition to disruption of haemostasis. Damaged endothelial cells are unable to contain macromolecules and fluid in the circulation, and there is a dramatic reduction in blood volume and a corresponding increase in haematocrit (91). Nevertheless, after a critical period of 24–36 h, vascular function is restored and a rapid return to homeostasis ensues (91). This vascular leak appears to rely mainly on endothelial activation by the viral NS1 protein (92) via TLR4 (92). Interestingly, TLR4 is also the receptor for LPS, a bacterial endotoxin and a key driver of sepsis (93).

#### Kawasaki Disease

While children appear to be relatively resistant to the respiratory complications of COVID-19, SARS-CoV-2 has been linked with a recent rise in the occurrence of Kawasaki disease (94). Kawasaki disease is a rare multi-system inflammatory response syndrome and vasculitis. It occurs in young children and is believed to arise due to post-viral immune reactions. SARS-CoV-2-mediated Kawasaki disease appears to have several distinct features, including severe haemodynamic instability and associated myocarditis (95). Localised dermal vasculitis associated with intra-endothelial SARS-CoV-2 particles has also been reported in children (4). Interestingly, these children tested negative for the virus by standard qPCR, but retained SARS-CoV-2-like particles inside endothelial cells in lesional areas that also stained positive for SARS-CoV-2 spike protein (4).

## Endotheliotropism may Influence the Outcome of Viral Infection

### Endotheliotropism

**Figure 2:**
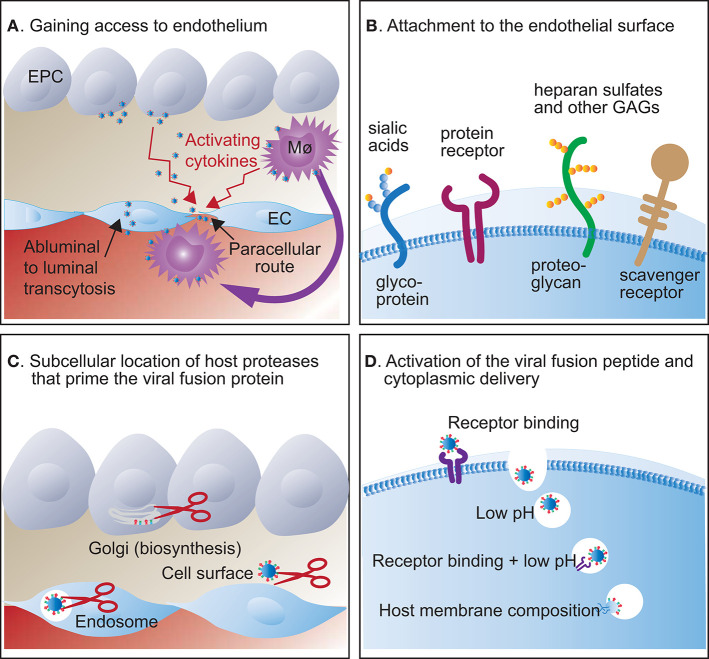
Host factors that regulate viral entry into endothelial cells**. (A)** After replication in mucosal epithelial cells (EPC), viruses can infect endothelial cells (EC) abluminally or gain access to the luminal surface by transcytosis, paracellular dissemination through activated endothelial junctions, or by being carried by cells that traffic between blood and tissues, like monocyte/macrophages (Mø). **(B)** Viruses attach to carbohydrate or protein molecules on the endothelial cell surface. Typically, attachment to protein receptors and sialic acid variants is highly specific, while attachment to heparan sulphates and scavenger receptors is more promiscuous. **(C)** The viral fusion protein is primed, typically by proteolytic cleavage, in the Golgi during biosynthesis, on the cell surface, or in endosomes, depending on protease susceptibility. **(D)** Further activation of the viral fusion peptide and viral cytoplasmic delivery may happen on the cell surface or in endosomes and depends on a conformational change triggered by receptor binding and/or specific pH requirements. Some viruses only fuse with host membranes of specific compositions.

The tropism of a pathogen is the host, cell type or tissue that supports its growth and, in the context of a virus, the generation of new progeny. Hence, an endotheliotropic virus is defined by its ability to attach to, enter, and replicate in endothelial cells. Such tropism depends on several factors (summarised in Figure 2) that will be discussed in detail in section 2.10. For viruses like SARS-CoV-2 (2), henipavirus (9), hantavirus (11), HPAIV (12), and ISAV (13), examination of tissues from infected individuals provides strong evidence of endotheliotropism (Table 1). The observation of intracellular virus particles by electron microscopy, endothelial expression of viral proteins by immunostaining, and/or endothelial viral RNA production by *in situ* hybridisation shows that endothelial cells are targeted during infection. Importantly, the specificity of reagents detecting viral antigens and nucleotides can be ensured by including tissues from non-infected individuals. Single-cell RNA-sequencing and analysis of enriched cell populations from digested tissues can also be used to map the presence of virus in individual cell types, again with a high specificity. However, sensitivity is probably a limiting factor of all these techniques, as cells with a low viral load may evade detection. Furthermore, neither of the techniques discussed above provide definitive evidence of generation of infective viral particles, although the observation of budding viral particles by electron microscopy is a strong indication. To evaluate whether infective progeny is produced requires serial passaging of virus, most often in cell culture. Conversely, the ability to replicate in cultured endothelial cells does not prove that the virus is endotheliotropic during natural infection. For example, while both Zikavirus and West Nile virus readily replicates in cultured endothelial cells (96, 97), endothelial expression of viral antigens does not appear to be a prominent feature of human infection.

### Challenges Associated With Studying Endothelial Viral Infections

The discrepancy between observations of replication in cell culture and the lack of endothelial expression of viral antigens during natural Zikavirus and West Nile virus infections, underscores a highly relevant challenge. Often, detailed insight in the molecular processes of viral infection requires work in cellular model systems. Because many phenotypic traits of endothelial cells rely on input from their surroundings, endothelial cells drift when they are removed from their natural microenvironment (25). Hence, cultured endothelial cells may show a different repertoire of surface proteins and other modulators of viral infection than endothelial cells in tissues, leading to different outcomes. Moreover, culture systems do not reflect the complex interplay between stromal and immune cells that makes up the host defence to infection. Hence, the design and interpretation of cell culture experiments should always be done with a view to patient material analyses. However, working with patient samples also imposes challenges. For highly pathogenic and contagious agents, the proportion of deceased patients that undergo a full autopsy may be low and could lead to bias. Moreover, analyses of unfixed tissues must be performed under high-level biosecurity restrictions, imposing substantially increased costs and limited availability of specialised equipment. Another challenge is that endothelial cells reside in tissues. Hence, sampling of material from human infections commonly originates from the terminal stage of the disease and may be poorly suited to study early events or even the peak viremic phase of infection. However, endothelial function can be evaluated non-invasively in patients by FMD (see section 2.3.1), making it possible to study viral effects on endothelial function at all stages of infection. Experimental infection of laboratory animals is often used to help our understanding of infection and disease development, including the sequential infection of specific cell populations. Because the pathogenesis of disease often differs between species, animal models of human viral infections have important limitations. Hence, the study of animal viruses in their natural hosts may have an important complementary role in studying host-pathogen interactions. In conclusion, several approaches should be used to determine the target cell-type repertoire of a virus. Moreover, while the use of model systems is essential to obtain information at high resolution, results should always be interpreted in the context of observations from natural infections.

### The Ability to Infect Endothelial Cells Is Associated With Virulence

For some viruses, the conversion from a well-tolerated to a virulent phenotype involves modulations of viral surface proteins that make the virus able to infect endothelial cells. Well-studied examples include avian influenza viruses of the H5 and H7 subtypes and ISAV, all belonging to the orthomyxovirus family. While low-pathogenic avian influenza virus (LPAIV) only replicates in mucosal epithelial cells, the conversion of H5 and H7 strains that defines HPAIV is associated with a switch in cellular tropism (12, 98). Typically, infection of susceptible bird species (domestic poultry and black swans) with HPAIV results in a global vascular infection pattern (12, 99, 100), where immunostaining of viral antigens gives a stronger signal in endothelial cells than in epithelial cells of the same organ (100). Accordingly, HPAIV strains efficiently replicate in cultured human endothelial cells (101). One should note that viral replication in endothelial cells does not appear to be a prominent feature of influenzavirus infection in humans and other mammals (12, 102). Nevertheless, the introduction of a miR-126 binding site that specifically prevented viral replication in endothelial cells, restricted H5N1 replication to the lung in experimentally infected mice and ameliorated disease in both mice and ferrets (103). In contrast, preventing viral replication in haematopoietic cells did not affect disease development (103). While only coming out of a single study, these findings suggest that the ability to infect endothelium may play a role in the extra-pulmonary dissemination and development of generalised H5N1 infection, also in mammals. Interestingly, single-cell RNA-sequencing of mouse lung after infection with the pandemic 2009 H1N1 influenzavirus revealed viral mRNA transcription in 40–50% of endothelial cells (104). Nevertheless, the level of transcription in individual endothelial cells was low, raising the question of whether it would be detected by conventional methods like immunohistochemistry and *in situ* hybridisation. Like avian influenza virus, ISAV exists in two variants. Similar to LPAIV, the ubiquitous non-pathogenic HPR0 variant only infects epithelial cells at mucosal surfaces and cannot invade the interior of the fish (105). However, specific modulations of surface glycoproteins induce a dramatic increase in ISAV pathogenicity accompanied by a switch in cellular tropism and multi-organ infection of vascular endothelial cells (13). Together, these observations suggest that the ability to infect endothelium may be a critical factor in supporting generalised viral dissemination and promote disease.

The routes by which viruses reach endothelial cells during natural infection (Figure 2A) have not been completely mapped. One interesting question is the extent to which endothelial cells are infected from the abluminal tissue surface (Figure 2A). Considering a model where endothelial infection facilitates viral spread from mucosal sites to the circulation, viral endothelial entry would be expected to be abluminal. While some virus receptors, like integrins (106), are abundantly expressed on both abluminal and luminal endothelial surfaces, both influenzavirus (107–109) and ISAV (13, 110) attach to cells by binding sialic acids that are mainly expressed in the glycocalyx on the luminal surface of endothelial cells (42). Furthermore, work in polarised endothelial cultures shows that HPAIV infects cells most efficiently from the luminal side (111). However, to infect endothelium from the luminal cell surface, the virus must cross the vascular barrier by other means. These could include abluminal-luminal endothelial transcytosis or diffusion across intercellular junctions, perhaps promoted by inflammation or injury that increases vascular permeability. Moreover, trafficking cells may carry virus between peripheral tissues and the circulation [discussed in (112)]. An excellent example of the latter is Nipah virus that attaches to lymphocytes that do not support its replication, but act as carriers and promote endothelial trans-infection (113). Interestingly, budding of H5N1 and ISAV mainly occurs from the luminal endothelial surface (13, 99, 114), suggesting that endothelial-produced viral particles, at least for these viruses, predominantly enter the circulation rather than being released to surrounding tissues (115).

It should be noted that many viruses cause devastating pathology at the site of initial infection even in the absence of spread to distant anatomical sites, exemplified by the extensive pulmonary damage caused by SARS-CoV-2 and seasonal human influenza. Hence, endotheliotropism is by no means the only or even the major determinant of virulence.

### The Ability to Cross Specific Vascular Beds Contributes to Viral Tissue-Tropism

Infection of endothelial cells may also allow circulating virus particles to enter tissues, provided that the virus can bud from or be transcytosed to the abluminal endothelial surface. Again, alternative routes exist to enter tissues from the blood stream, including diffusion through activated or compromised endothelium, endothelial transcytosis, or a Trojan-horse approach relying on infection of cells that traffic between blood and tissues (112). The relative importance of these pathways remains unclear.

Arthropod-borne viruses of the flavivirus family, like Zikavirus and West Nile virus, transmit by mosquito or tick bites and gain more or less direct access to the circulation. These viruses provide interesting examples of how circulating viruses target specific organs and give rise to distinct patterns of disease. Both viruses primarily cause pathology in the central nervous system, thus, the ability to cross the blood-brain barrier is central to pathogenesis. In addition, the ability to cross the placenta is highly relevant to Zikavirus pathology, where severe viral damage is restricted to the foetus. In the early phase of infection, the affinity for endothelial surface receptors appears to be a key factor in determining these agents' tissue-tropism (96, 116, 117). So far, there is limited evidence that Zikavirus and West Nile virus replicate in endothelial cells during natural infection. Their ability to cross intact endothelium probably relies on properties that allow attachment and transcytosis across the intact endothelial barrier, rather than productive infection (116, 117). Moreover, when the infection progresses, and inflammatory responses accelerate, increased vascular permeability appears to promote viral egress further, exemplified by the peripheral TLR3-dependent inflammation that facilitates West Nile virus entry into the brain (118). Also, several flaviviruses, including Zikavirus and West Nile virus, selectively disrupt the endothelial glycocalyx of their target tissues via secreted viral proteins, resulting in increased tissue-specific vascular permeability (119) that may also facilitate the entry of circulating viral particles.

### Could Endothelial Infection Augment Viral Amplification?

The importance of viral replication in endothelial cells remains less clear. Nevertheless, the amelioration of disease in mouse and ferret models of H5N1 influenza upon endothelial-specific inhibition of viral replication (103) suggests that replication in endothelial cells at least in some cases promotes a more severe disease. Considering the abundance of endothelial cells in the human body, estimated to make up ~20% of the total number of nucleated cells (20), we propose that viral amplification in endothelial cells deserves further study.

Altogether, there is substantial evidence that the ability to attach to, infect and/or travel across endothelial cells by transcytosis contributes to dissemination of viral infection and disease severity (42).

## Specific Factors That Determine the Ability to Infect Endothelial Cells

### Viral Attachment to Endothelial Cells Ranges From High to Low Specificity

The first step of the infectious cycle is the attachment of virus particles to the host target cell membrane. This process is mediated by interactions between viral surface glycoproteins and their corresponding cellular receptors (107, 120, 121). The endothelial glycocalyx presents a range of molecules involved in viral attachment and entry (Figure 2B). The binding of viral glycoproteins to such glycan and protein receptors is often highly specific and contributes to determining cellular and host specificity (122), although the presence of a viral receptor is not sufficient to allow infection. Patterns of virus binding can be mapped directly by virus binding assays [e.g., (13, 123)] or indirectly by mapping the expression of specific viral receptors by immunostaining. Lately, large datasets from single-cell RNA-sequencing experiments have been used to map the expression of receptors and proteins involved in SARS-CoV-2 attachment and entry by different cellular subsets (124, 125). Nevertheless, information from such analyses must be considered with the reservation that the correlation between mRNA and protein expression depends on several factors, including posttranslational modifications and protein half-life (125, 126). It should also be kept in mind that even when expressed at low levels, receptors may still be present in sufficient amounts to support viral entry. Hence, the expression level of a viral receptor should be regarded in the context of its binding affinity for the virus attachment glycoprotein. While low-affinity binding, as observed between influenzavirus hemagglutinin (HA) and sialic acids, requires the abundant presence of the receptor for a high avidity association, a virus that binds its receptor with high affinity may require much lower expression levels of the receptor to support robust attachment (120).

Coronaviruses attach to host cells by their surface-exposed spike (S) glycoprotein that forms the characteristic crown shape that gave name to this virus family (127). The S protein consists of two non-covalently attached subunits, the S1 and S2, responsible for attachment and fusion, respectively (19). The N-terminal and C-terminal domains of the coronavirus S1-protein bind carbohydrate and protein receptors, respectively, and coronaviruses vary widely in their choice of attachment receptors, including both *O*-acetylated and non-acetylated sialic acids and proteins (19, 127). The SARS-CoV-2 S1 protein predominantly binds the protein ACE2 (35–37), although binding to 9-*O*-acetylated sialic acids (128, 129) and CD147 (cluster of differentiation 147) (130) has also been reported. In addition to strong surface expression in alveolar epithelial cells and enterocytes of the small intestine, ACE2 is expressed by arterial and venous endothelial cells and arteriolar smooth muscle cells (126). When SARS-CoV-2 S1 binds ACE2, the spike trimer opens up and unshields the S2 core, thus facilitating fusion protein activation (131).

Henipaviruses (i.e., Nipah and Hendra virus) provide another example of viral use of a protein receptor for initial cellular attachment. Henipavirus G proteins specifically bind the vascular endothelial tyrosine kinase EphrinB2 (120, 132). Nipah virus also binds EphrinB3, which is expressed to a lesser degree on endothelial cells, but is highly expressed in the central nervous system (120). Hantaviral attachment and entry receptors are associated with more uncertainty, with β3-integrin, gC1qR, and protocadherin-1 all being proposed as candidates, possibly with complementary roles (133). Interestingly, attachment to protocadherin-1 appears to be a clade-specific trait of new world hantaviruses. The receptor is mainly expressed on airway endothelial cells, correlating to the pathology mediated by this clade, which is the causal agent of hantavirus pulmonary syndrome (133).

Virus binding to sialic acids has been extensively studied in the context of influenza A. Sialic acids are found on the outermost end of glycan chains of most cells, but are particularly abundant in the thick luminal glycocalyx layer covering all luminal endothelial surfaces (42). Here, they maintain a negative net charge and promote the vascular barrier function (44). For influenzavirus, the specificity of binding is regulated by the nature of the sialic acid linkage to underlying sugars. The ratio and distribution between 2,3-linked and 2,6-linked sialic acids in the airways vary greatly between species, and the potential for airborne transmission within specific host populations is primarily determined by whether HA preferentially targets 2,3-linked or 2,6-linked sialic acids (134, 135) [discussed in (108)]. Other viral glycoproteins attach to specific sialic acid derivatives. For example, ISAV hemagglutinin esterase (HE) exclusively binds 4-*O*-acetylated sialic acids (110, 136) expressed by endothelial, some epithelial, and red blood cells of a range of vertebrates (137). It is important to realise that while successful viral attachment is a requirement, it may not be sufficient to mediate viral infection. For example, the LPAIV strains H5N9 and H6N1 match the HA binding pattern of the endotheliotropic HPAIV H5N1 (123), suggesting that both LPAIV and HPAIV can attach to endothelial cells. Similarly, non-pathogenic epithelial-restricted and pathogenic endotheliotropic ISAV HE variants have similar receptor-binding properties (138).

Virus particles may also attach non-specifically to cells via interactions with carbohydrates and/or scavenger receptors. Such attachment strategies are particularly frequent among the flavi- and filoviruses that typically target a broad range of cells and appear more promiscuous in their choice of entry receptors. Subsets of endothelial cells express scavenger receptors that facilitate the removal of waste macromolecules and apoptotic cells from blood (24, 139). Stressed virus-infected cells are prone to exposing the scavenging-signal phosphatidyl serine on their outer surface, resulting in frequent exposure of phosphatidyl serine on the outside of the cell-derived viral envelopes (140). Viral phosphatidylserines facilitate the attachment and uptake of dengue virus via scavenging receptors, either directly (TIM family receptors), or indirectly via the natural phosphatidylserine receptors GAS6 and PROS (TAM family receptor ligands) (23). Zikavirus appears to have a particularly high affinity for GAS6, providing a possible explanation for its specific ability to attach to foetal endothelial cells via the TAM receptor AXL, cross the placenta, and cause pathology in the foetus (96). Another example of a non-specific strategy is the initial attachment of ebolavirus, where uptake is facilitated by scavenger receptors like TIM-1 (141) and C-type lectins (142), the latter expressed by hepatic sinusoidal endothelial cells (142, 143). Non-specific interactions with glycosaminoglycans may also serve as a primary anchoring step that promotes subsequent engagement with a specific protein receptor. While not an emerging virus, endotheliotropic Kaposi's sarcoma herpes virus (KSHV) is a good example of this. KSHV attaches to heparan sulphate glycosaminoglycans before its subsequent specific engagement of the protein co-receptors EphrinA2 and DC-sign. Together, this attachment initiates signalling that allows fusion and cellular entry (144). DC-sign homologues are also thought to mediate the attachment of other viruses to endothelium, including dengue (145), Ebola (142), and hepatitis C virus (143).

In conclusion, viral attachment to endothelium occurs both by highly specific interactions with cellular receptors and less specific interactions with scavenging receptors or charged molecules like surface glycosaminoglycans. Binding to endothelial cell surfaces is required for successful infection and, as demonstrated by new world hantaviruses, may be linked to organ-specific pathology. Nevertheless, additional factors are needed to permit endothelial infection, as demonstrated by HPAIV and ISAV.

### Access to the Endothelial Cytoplasm Relies on Viral Fusion With the Host Cell Membrane

In the next step of the infectious cycle, the virus gains access to the cellular interior. This step is essential for determining cell tropism. For enveloped viruses, viral entry happens when the viral envelope fuses with the cell membrane and releases its contents to the cytoplasm (146). Viral fusion proteins are activated in a two-step process that results in exposure of a hydrophobic fusion peptide that can be inserted into the host cell membrane to start the fusion process. This peptide may consist of mainly α-helices (class I), mainly β-sheets (class II), or a mixture of both (class III) (146). Viruses discussed here contain class I or class II fusion proteins, listed in Table 3.

#### The Availability of Relevant Proteases Regulates the Priming of the Viral Fusion Protein

Before fusion can be triggered, class I and class II fusion proteins must be primed to reach a fusion-competent state, with the possible exception of hantavirus Gc protein (147). Priming usually involves a proteolytic cleavage that may take place in different subcellular compartments or externally. Accordingly, viral fusion proteins may be primed in the Golgi during viral biosynthesis, externally at the cell surface, or in endosomal compartments (Figure 2C), depending on both intrinsic properties of the fusion peptide and the range of host proteases present (146). Fusion protein priming plays an essential role in determining cellular tropism, as many proteases are expressed in a tissue-specific manner (148). For example, HA molecules of seasonal influenzavirus are predominantly cleaved by airway proteases, hence, priming happens on the epithelial cell surface [discussed in (149)]. This is in contrast to avian H5 and H7 influenzaviruses, where the conversion to HPAIV involves the acquisition of multiple basic amino acids at the HA cleavage site (98). This makes the site susceptible to an extended range of proteases, including ubiquitous pro-protein convertases like furin that are encountered in the Golgi apparatus during viral biosynthesis (150). Thus, susceptibility to pro-protein convertase cleavage allows the budding of virus particles that express pre-primed HA molecules and can infect a much wider range of cells than those depending on priming by tissue-restricted cell surface proteases.

A pro-protein convertase cleavage site at the S1-S2 position of the coronavirus S protein also appears to mediate extended cellular tropism. This cleavage site is present in Mediterranean respiratory syndrome coronavirus (MERS-CoV), but not in 2003 SARS-CoV. Accordingly, budding MERS-CoV viral particles express cleaved S proteins, while SARS-CoV viral particles predominantly express S proteins in the uncleaved immature conformation (151). Like MERS-CoV, SARS-CoV-2 has acquired a pro-protein convertase site at the S1-S2 junction that allows cleavage of the S protein during viral biosynthesis (35, 152, 153) and increases the efficiency of viral fusion with the plasma membrane (153). Interestingly, protein cleavage by furin may also expose a C-terminal R/KXXR/K-motif that strongly promotes cellular internalisation by interaction with the cellular receptor neuropilin-1 (154). This is also the case for SARS-CoV-2. Furin-mediated S1-S2 cleavage generates a SARS-CoV-2 S1 peptide with a C-terminal amino acid sequence (^682^RRAR^685^) that binds neuropilin-1, which acts as a co-factor to promote viral entry and infection (155, 156). The significance of furin cleavage to endothelial tropism has not been directly addressed, but neuropilin-1 is strongly expressed in endothelial cells in pulmonary tissues (156). Moreover, it is reasonable to hypothesise that a lesser dependency on surface-expressed proteases may extend the cellular tropism of SARS-CoV-2 and could contribute to its ability to infect endothelial cells.

Finally, the henipavirus F protein is an example of a viral fusion protein that is primed in the endosome, where it is cleaved by the endosomal protease cathepsin L (157, 158).

#### The Microenvironment That Triggers Fusion and the Subcellular Location Where It Is Encountered Vary Between Viruses

After priming, the triggering of fusion is initiated by a conformational change that exposes the fusion peptide (Figure 2D). This conformational change can happen in response to a single event, often the binding to a specific receptor or the exposure to low pH. Alternatively, it may require a series of activating events, like receptor binding followed by exposure to low pH and/or proteolytic cleavage. Fusion proteins that depend on receptor binding only to trigger their activation, generally fuse with the host cell membrane at the cell surface (146). The previously mentioned KSHV belongs to this category, as do most other herpesviruses. This may be relevant when considering endothelial cell tropism, as some studies suggest that endothelial endosomes resist fusion of certain viruses, as discussed below.

A large proportion of enveloped viruses enters the endocytic pathway before fusion, travelling through endosomal and lysosomal compartments until they encounter a microenvironment that provides optimal conditions for triggering fusion (146). Influenzavirus HA belongs to the category of fusion proteins triggered by exposure to low pH alone. Interestingly, human lung microvascular endothelial cells show less efficient endosomal acidification than human epithelial cells (159), and this renders endothelial cells less permissive to infection by seasonal influenzavirus and LPAIV strains. In addition, endothelial cells express high constitutive levels of antiviral interferon-induced transmembrane protein (IFITM) 3 in endosomal and lysosomal compartments. This protein arrests endosomal fusion of seasonal human influenzavirus in endothelial cells at the hemifusion stage (160). Overcoming these obstacles, HPAIV strains have a less stable conformation of the HA molecule that allows fusion to take place at a higher pH (159) and allows infection in early endosomal compartments. The ability to efficiently fuse at a higher pH probably also mediates the relative resistance of HPAIV to the antiviral action of IFITM3 (161). Notably, it is unknown whether the inefficient endosomal acidification and high expression of IFITM3 are specific to the lung microvasculature or a general feature of endothelial cells. The relevance of these findings to other anatomical sites is therefore unclear. Furthermore, the efficiency with which IFITM proteins inhibit viral entry vary between viruses. Flaviviruses, represented by dengue and West Nile virus, show a similar susceptibility to IFITM proteins as influenza H1N1, while arenaviruses appear to overcome their action (162).

In viruses where separate viral glycoproteins mediate cellular binding and fusion, interactions between these proteins may also regulate viral entry. This has been extensively studied in paramyxoviruses, where three different models have been proposed: First, the *provocateur model*, where viral binding to cellular receptors, often sialic acids, triggers an activating interaction between the viral attachment and fusion proteins; Second, the *clamp model*, where the interaction between the viral attachment and fusion proteins inhibits the fusion protein; And third, the *safety-catch model*, where the fusion protein head tightly assembles with the stalk protein of the attachment viral glycoprotein in the endoplasmic reticulum during viral bio-synthesis, but is released at the target cell membrane upon receptor binding (163). A highly relevant example of the safety-catch model is the binding of endotheliotropic henipaviruses, where binding of the globular head of dimeric G-proteins to their cellular receptors (EphrinB2 and B3) mediates a distant structural change that releases the association with the F-protein, thus allowing fusion to be triggered (163).

The ISAV fusion process is still incompletely characterised, but appears to have features in common with both influenza- and paramyxoviruses. For the F protein to reach its fusion-competent trimeric state, both low pH and proteolytic cleavage is required (164). The conversion to virulence that includes a gain of endotheliotropism, always involves deletion of a variable number of amino acids in a highly polymorphic region [HPR, including amino acids 320–374 (165)] at the base of the stalk of the hemagglutinin esterase molecule (166, 167). In apparent homology to the destabilisation of influenza HA that is associated with the conversion of LPAIV to HPAIV, the shortening of the stalk that results from HPR-deletion destabilises the interaction with the fusion protein. This destabilisation appears to facilitate the dissociation of HE and F proteins upon receptor binding and allow activation of viral fusion at neutral pH (166, 168). In contrast, no increase in fusion efficiency was observed when comparing pathogenic and non-pathogenic variants of the HE and F proteins in the presence the exogenous protease trypsin and low pH (168), perhaps mimicking the situation at mucosal surfaces.

Exposure of the fusion peptide by receptor binding followed by protease cleavage, sometimes with the added requirement for low pH, has been studied extensively in coronaviruses and is therefore of great relevance to the current pandemic. Endothelial cells express a wide range of proteases on their luminal surface that take part in activation of surface receptors with homeostatic functions. As discussed above, the SARS-CoV-2 S protein is cleaved during biosynthesis by pro-protein convertases. However, further S-protein processing occurs at the target cell surface by the protease TMPRSS2 and in target cell lysosomes by cathepsin, and the three proteases appear to have cumulative effects in regulating the entry of SARS-CoV-2 (153). In contrast to cathepsins that are ubiquitously expressed in lysosomes, TMPRSS2 expression differs between tissues and has been proposed to restrict the target cell population of SARS-CoV-2 (169). While TMPRSS2 is strongly expressed by human endothelial cells in culture (124), only a minor fraction of tissue endothelial cells appears to express the TMPRSS2 gene under basal conditions (125, 170, 171), perhaps limiting endothelial SARS-CoV-2 infection. Yet, endothelial cells are infected (2, 3), presumably assisted by the additive action of pro-protein convertases and cathepsins.

The composition of the host cell membrane may also regulate the potential for viral fusion. A prime example is the Andes hantavirus that shows a strict requirement for membrane cholesterol that is shared by several other pathogenic hantaviruses (172). Similarly, dengue virus requires anionic lipids in the host cell membrane and therefore fuse in later endosomal compartments than their pH-requirements would indicate (173).

### Replication in Endothelial Cells Requires Evasion of Intrinsic Antiviral Responses

Once inside the cell, efficient viral replication depends on adaptation to the host cell machinery. The ability of viral polymerase complexes to function efficiently in the cellular interior is a key factor for determining species tropism and virulence, as demonstrated for PB2 and PA subunits of influenza (174, 175). Moreover, viral polymerase subunits must be able to associate with the proteins that mediate nuclear import in the cell type in question (176). Nevertheless, successful viral adaptation to the endothelial cell type may rely even more on the ability to evade intracellular antiviral responses that vary between cell types. For example, replication of pathogenic hantavirus was sustained in cultured endothelial cells for up to 48 h, while infection with a non-pathogenic strain resulted in rapid upregulation of antiviral proteins that prevented efficient generation of infective progeny (177). This correlates to observations in ISAV infection, where a pathogenic isolate of low virulence infected endothelial cells early in the course of infection, but was rapidly eliminated, while a high-virulent pathogenic isolate that caused a less pronounced antiviral response, maintained replication in endothelial cells over a prolonged period of time (178, 179). In conclusion, the efficiency of viral replication in any cell type relies on successful viral adaptation to the cellular replication machinery as well as the ability to evade intrinsic antiviral responses.

## The Endothelial Cell Response to Viral Infection

### Cellular Detection of Viral Components Activates NF-kB and Type I Interferon Signalling

All cells are capable of intrinsic antiviral responses. First, cellular pattern recognition receptors at different subcellular sites detect the presence of viral nucleic acids. The Toll-like receptors TLR7 and TLR8 (both detecting RNA), and TLR9 (detecting DNA) bind viral nucleotides on cell surfaces or in endosomes. Once released to the cell interior, viral contents can also be detected by the cytoplasmic receptors RIG-like (e.g., retinoic acid-inducible gene-I, RIG-I, detecting single stranded RNA), melanoma differentiation-associated protein 5 (MDA5, detecting double stranded RNA), and cyclic GMP-AMP synthase (cGAS, detecting DNA). Recent work suggests that the surface-expressed receptor TLR4 can also be activated by secreted viral proteins (92, 180). Stimulation of these receptors initiates signalling that results in the intracellular activation of inflammatory (NF-kB) and type I interferon (interferon response factors 3 and 7) transcription factors (181). Similar to other cell types, endothelial cells mount type I interferon responses upon viral infection (177, 182). This response stimulates the transcription of a range of interferon-stimulated genes (ISG) that inhibit viral infection (183). Furthermore, endothelial activation of NF-kB results in upregulation of a range of molecules that mediate leukocyte adhesion and shift the endothelial surface toward a pro-thrombotic stage (184) (Figure 1C).

### Inflammatory and Antiviral Signalling Is Also Activated in Non-infected Cells

Non-infected cells, including endothelial cells, also respond to viral infection (104). Endothelial cells express receptors for type I and II interferons, as well as a range of cytokines and chemokines upregulated in and secreted from virus-infected cells or activated immune cells (Figure 1C). Such activation is highly prevalent in patients with H1N1 2009 pandemic influenza and COVID-19 (81, 90). Another example of such humoral activation is the vasoactive proteins secreted by dendritic cells and macrophages in ebolavirus infection (180). In addition, non-infected cells can be activated in viral infection by diffusion of second messengers across intercellular gap junctions (185). Although only demonstrated for the cGAS second messenger cGAMP(2',5'), it is likely that other low molecular weight (<1,000 Dalton) second messengers also share this route (186). Gap junction-mediated activation of endothelial cells has not been explored in the context of viral infection. However, the mechanisms could be highly relevant, as endothelial cells form gap junctions with other endothelial cells, vascular smooth muscle cells, and pericytes that contribute to the maintenance of vascular homeostasis (68, 69).

### Endothelial Cells Are Effectors of the Host Response to Viral Infection

Endothelial NF-kB activation is capable of mediating all cardinal signs of inflammation. Redness, swelling, heat, and pain result from an increase in local blood flow, leakage of protein-rich plasma, and the recruitment of leukocytes that release mediators working on C-type sensory nerve fibres. Invading leukocytes attempt to eliminate the initiating stimulus by killing infected cells and removing cellular debris. However, the powerful inflammatory mediators released by leukocytes also exacerbate vascular activation, cause tissue damage, and loss of function (Figures 1C,D). The first cells recruited to sites of inflammation are neutrophils that infiltrate tissues within minutes after endothelial type I activation (53). Nevertheless, a recent study showed that recruitment of non-conventional CD8 αβ-T cells from the microvasculature occurred with similar ultra-rapid dynamics when a rhabdovirus was inoculated in the nasal cavity of trout, and that this recruitment was central to controlling infection (187). The mechanisms involved are not yet fully understood, but appear to depend on viral activation of tyrosine kinase receptors (187). Altogether, the dynamic vascular expression of adhesion molecules and chemokines in inflammation contributes to tight coordination of the leukocyte recruitment process. Such control is particularly relevant in viral infection, where pathology often arises from the host response to infection, rather than the action of the virus itself. The importance of controlling early innate responses and preventing their spiralling out of control is clearly demonstrated in COVID-19, where an increased neutrophil-lymphocyte ratio is a feature of severe disease and has emerged as a prognostic indicator of outcome (188).

### Endothelial Cells Modulate Immune Cell Activation in Viral Infection

Infection with some endotheliotropic viruses, like SARS-CoV-2 (2) and Nipah (9, 189), is associated with extensive endothelial damage and endotheliitis (Table 1). In contrast, other endotheliotropic infections are characterised by a lack of perivascular cellular infiltrates and/or endothelial cytopathology (11, 13). This suggests suppression of cytotoxic T cell responses, which would otherwise attempt to limit infection by killing infected cells. In this context, it is interesting to consider the immunomodulatory potential of antigen-presenting endothelial cells. As part of protective measures against self-reactive T cell responses, interferon-induced MHC class II allows endothelial cells to act as semi-professional antigen-presenting cells (190). Importantly, type I interferon signalling also upregulates programmed cell death 1 (PD-1) ligand (PD-L1) in endothelial cells (191), and T cell activation by PD-L1-bearing lymphatic endothelium induces accumulation of PD-1 and T cell deletion (192). Hence, endothelial viral antigen presentation is likely to promote immune tolerance to protect vascular integrity, but also provide a niche for viral evasion of immune responses (41). Interestingly, PD-L1 on both haematopoietic and non-haematopoietic cells reduced viral clearance in experimental LMCV infection, yet improved survival by reducing immunopathology (193). There has been a recent discussion about the possible impact of PD-1 blockade, a commonly used cancer immunotherapeutic, on SARS-CoV-2 infection. Nevertheless, current clinical data suggest that it does not change the outcome in COVID-19 (194).

## Molecular Mechanisms That Disrupt Vascular Function in Viral Infection

### Maintaining a Stable Vasculature Is Key to Preventing Viral Pathology

**Figure 3:**
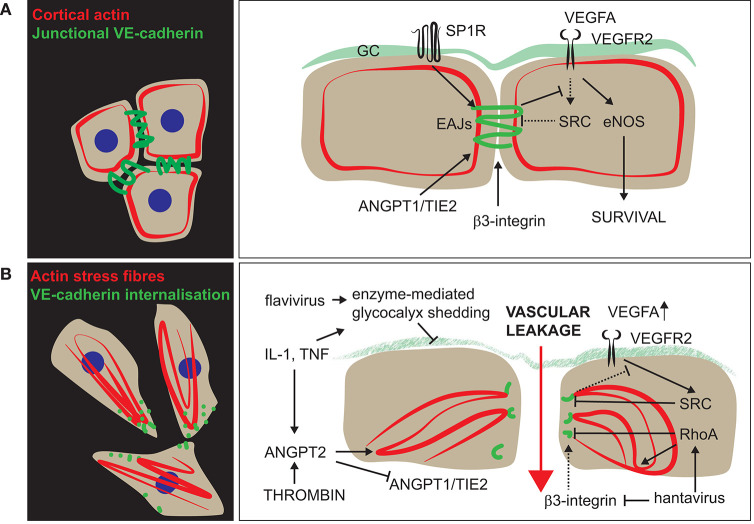
Functional regulation of vascular permeability in viral disease. The vascular barrier integrity is controlled by two main components: the glycocalyx (GC) and the endothelial adherens junctions (EAJs). **(A)** In the resting state, several signalling pathways promote VE-cadherin-supported junctional integrity. In addition to maintaining an intact vascular barrier, VE-cadherin-signalling also skews VEGFR2-signalling toward eNOS-activation and survival-promoting pathways. **(B)** Upon type I (thrombin) and/or type II (IL-1, TNF) activation, the cytoskeleton is reorganised, and fibrillar adhesions are formed in an ANGPT2-dependent manner. Furthermore, activation by cytokines or flaviviral NS1 stimulates endothelial production of enzymes that mediate glycocalyx-shedding and further disrupts the vascular barrier. Hantavirus surface proteins bind and inactivate the EAJ-supportive action of β3-integrins, thereby increasing VEGF-stimulated permeability. In addition, hantavirus N protein promotes RhoA-signalling and increases permeability independent of VEGF-signalling.

Studies of the S1P-receptor illustrate the central role endothelial cells have in maintaining the vascular barrier and preventing detrimental inflammation. Activation of endothelial S1P-signalling by a synthetic agonist suppressed early innate immune responses and improved survival in mice infected with H1N1 2009 pandemic influenzavirus (83). S1P-administration also ameliorated hantavirus-induced endothelial hyper-responsiveness in a cell-based permeability assay (195), showing that S1P-signalling has a generalised stabilising effect on the vasculature. Underscoring the importance of endothelial signalling in maintaining a functional vasculature, mice with endothelial-specific deletion of the glucocorticoid receptor showed increased mortality in systemic inflammatory response syndrome, with exaggerated vascular instability and pro-inflammatory signalling (196). This makes it tempting to speculate if some of the positive effects of glucocorticoid-treatment in severe COVID-19 (197) could be mediated by vascular stabilisation. An overview of the molecular mechanisms that regulate the stability of vascular junctions in viral disease is provided in Figure 3.

### Disruption of Vascular Function by Host Defence Mechanisms

#### Excessive NF-kB and Type I Interferon Signalling Disrupts Endothelial Function

Signalling that confers powerful antiviral actions also has less beneficial effects (Figure 1). Type I interferon responses are associated with endothelial dysfunction in systemic lupus erythematosus (198). They also accelerate the development of atherosclerosis in both mice and humans, at least in part by reducing NO bioavailability [reviewed in (199)]. Finally, type I interferon skews the profile of endothelial-secreted molecules toward a pro-thrombotic state and inhibit normal angiogenic functions that may be relevant to reparatory processes (200). Many ISGs have dual, counteractive functions, a highly relevant example being PAI-I (201). PAI-I is an extracellular serine protease inhibitor that counteracts viral entry by inhibiting tryptase, trypsin, and TMPRSS2, thereby inhibiting priming of influenzavirus HA (201). However, PAI-I is best known for its pro-thrombotic actions and is a biomarker for endothelial dysfunction (202). Hence, excessive or prolonged endothelial secretion of PAI-I will disturb the delicate maintenance of blood fluidity (Figure 1D).

Similarly, excessive endothelial NF-kB-activation is linked to the disruption of normal vascular function. Stimulation of NF-kB promotes a pro-inflammatory and pro-thrombotic endothelial phenotype (184) and inhibits NO production (203). Accordingly, endothelial-specific deletion or inhibition of NF-kB protects mice from developing atherosclerosis (204) and abrogates the derangement of vascular functions in experimental sepsis (203). In conclusion, prolonged or excessive endothelial activation in viral disease is likely to result in vascular dysfunction that affects both infected and bystander endothelial cells.

#### Mast Cell Activation Induces Vascular Hyper-Permeability

Host cells may also secrete other vasoactive molecules. One of many examples is the vascular hyper-permeability induced by vessel-associated mast cells in dengue-infection, involving tryptase-mediated breakdown of intercellular endothelial junctions (205). Tryptase levels correlated with the severity of symptoms in two patient cohorts (205), supporting that this mechanism is relevant to disease pathogenesis.

### Disruption of Vascular Function by Viral Components

#### Viral Surface Proteins That Dysregulate Vascular Homeostasis

Many of the cellular molecules that viruses bind (Table 2) are also central to the regulation of vascular function (209). Hence, viral infection has potential to dysregulate their signalling. In the COVID-19 pandemic, the dual roles of ACE2 have been subject to much attention (19, 210), on one hand serving as a key factor for viral attachment and entry (153), and on the other hand, counteracting the detrimental cardiovascular effects of ANG-II (19, 38), highly relevant to disease. Interestingly, internalisation of the closely related SARS-CoV is accompanied by internalisation and shedding of ACE2 (211, 212). By homology, it is broadly assumed that the same is true for SARS-CoV-2 (19, 210, 213). The resulting loss of the ACE2 catalytic effect at the cell membrane may be a significant contributor to pathology in COVID-19 (211, 213). Briefly, such downregulation of ACE2 favours the progression of inflammatory and thrombotic processes, triggered by enhanced and unopposed effects of ANG-II (19, 38, 213).

**Table 2:** Viral attachment molecules that bind endothelial surface molecules.

**Virus**	**Viral attachment protein**	**Endothelial receptor**	**References**
SARS-CoV-2	S protein, subunit S1	Angiotensin converting enzyme (ACE)-2	(35–37)
Nipah and Hendra henipavirus	G protein	EphrinB2 (B3)	(120, 132)
New world hantavirus	Gn protein	β3-integrin	(133)
		protocadherin-1	
Influenza A, H5N1, H7N1 (HPAIV)	HA, subunit H1	α-2-3-linked N-acetyl-sialic acid	(134, 135)
Infectious salmon anaemia virus (HPR-deleted)	HE	4-*O*-acetylated N-acetyl-sialic acid	(110, 206)

**Table 3:** Viral fusion proteins and factors that regulate their activation.

**Virus**	**Protein containing fusion peptide**	**Priming proteolytic cleavage (site)**	**Triggering**	**References**
SARS-CoV-2	S protein (S2 subunit)	Cleavage of S1 and S2 subunits that remain non-covalently bound (surface or endosome)	Proteolytic cleavage at the S_2_' site, immediately upstream of the fusion peptide	(35)
Nipah and Hendra henipavirus	F protein	Cleavage of F0 by cathepsin into F1 and F2 (endosome)	G protein receptor binding that mediates dissociation of the F protein	(157, 158, 163, 207)
New world hantavirus	Gc protein	Incompletely understood	Low pH, membrane cholesterol composition	(172)
Influenza A, H5N1, H7N1 (HPAIV)	HA, subunit H1	HA0 cleaved to HA1 (receptor binding) and HA2 (fusion), subunits remain non-covalently bound. (cell surface, endosome, Golgi)	Low pH	
Infectious salmon anaemia virus (HPR-deleted)	F protein	Cleavage of F0 by unknown protease into F1 and F2 (location not known)	Low pH, not fully characterised	(164, 208)

Another highly interesting SARS-CoV-2 interaction partner is the VEGFA- and semaphorin-binding b1 pocket of neuropilin-1 (155, 156). To our knowledge, the effects of this interaction on vascular permeability has not yet been evaluated in the context of SARS-CoV-2. Nevertheless, binding of C-end R/KAAR/K-peptides to this pocket generally increases vascular permeability in a VEGFR2-independent manner (214), and, upon injection, such peptides induce vascular leakage and are retained in tissues (154).

Another example of viral interactions with the VEGFR2-signalling pathway is the dysregulation of integrins in hantavirus pulmonary syndrome (Figure 3). Integrins have central functions in maintaining vascular barrier functions (215, 216), and β3-integrins specifically regulate VEGFR2-dependent vascular permeability (217). Pathogenic hantaviruses bind αvβ3 integrin in the bent, inactive conformation (218). The resulting reduction in αvβ3 integrin signalling results in VE-cadherin internalisation and increased vascular responsiveness to VEGF-A (195), mimicking the actions of αvβ3 integrin antagonism or genetic silencing of β3 integrin (57, 219) (Figure 3B).

EphrinB2, the cellular interaction partner for the henipavirus G protein (132), also modulates VEGFR2-signalling (220–222). Ephrins and Ephs are membrane-bound tyrosine kinases that signal bidirectionally. This leads to forward (in the Eph-bearing cell) and reverse (in the Ephrin-bearing cell) signalling in adjacent endothelial cells (223). EphrinB2-Eph4 signalling appears to support endothelial-pericyte interactions after cerebral ischaemic injury and promote the return to a functional vasculature (224). While EphrinB2-Eph4 signalling is dispensable for blood-endothelial barrier function, it is a critical stabiliser of lymphatic endothelial junction integrity (225). Therefore, it is tempting to speculate if viral EphrinB2 binding and internalisation could modulate EphrinB2-Eph4 signalling in henipavirus-infected endothelial cells. However, to our knowledge this has not been experimentally tested. The surface proteins of henipaviruses also mediate another central vasculopathy, namely the endothelial syncytia formation observed in both human (9) and hamster (189), a species commonly used to study the disease. This is reflected in cultured cells, where co-expression of henipavirus G and F proteins induces cellular fusion within 12 h (226).

Viral attachment to sialic acids also has the potential to affect vascular functions. In several viral infections, including influenza, a reduction in virus particle binding to host tissues is observed as the infection progresses (227), limiting superinfection (228). The mechanism behind the loss of virus binding is not fully characterised, but several studies suggest that sialic acid cleavage by viral neuraminidase may be involved (228–230). This is interesting, as sialic acids in the endothelial glycocalyx contribute to maintaining vascular integrity, and their removal has been associated with increased microvascular permeability (44). However, the consequences of virus-mediated sialic acid modulations on vascular function remain largely unknown.

#### Secreted Viral Proteins Damage Vascular Endothelial Cells Independent of Their Infection Status

Another virus family that targets the endothelial glycocalyx is the flavivirus family. An extensively studied example is the dengue virus-mediated vascular barrier breakdown that involves disruption of glycocalyx integrity in cell culture and animal models (231, 232). The damage is mediated by the secreted non-structural viral protein NS1. Dengue NS1 directly induces shedding of the pulmonary endothelial glycocalyx by upregulation of endothelial sialidases, cathepsin L, and heparanases (232) (Figure 3B). In fact, NS1 proteins of different flavivirus strains selectively target the endothelium of specific vascular beds in a pattern that corresponds to virus tropism and causes disruption of the endothelial glycocalyx and severe vascular leakage (119). At least for dengue virus, the capillary leak induced by NS1 is reduced by inhibition of TLR4 (92), suggesting that this receptor mediates the response to NS1. Interestingly, soluble viral glycoproteins appears to counteract the disruption of the vascular barrier caused by particle-bound viral glycoproteins in Ebola virus infection (233). Notably, infection of endothelial cells mostly occurs after clinical signs have developed in natural Ebola infection (16). Hence, most vascular damage is probably indirect, for example by the action of vasoactive cytokines secreted by dendritic cells and macrophages (180).

#### Modulation of Endothelial Signalling by Other Viral Proteins

The Andes hantavirus nucleocapsid (N) protein mediates an additional aspect of the increased microvascular permeability observed in hantavirus pulmonary syndrome (84). In contrast to disrupted integrin signalling that targets VEGF-stimulated vascular permeability (195), the N protein increases endothelial cell size by interfering with tuberous sclerosis complex inhibition of mTOR signalling (84). Because tuberous sclerosis complexes also regulate RhoA-signalling, the inhibition also activates RhoA and increases basal (unstimulated) vascular permeability (84) (Figure 3B).

In conclusion, both structural and non-structural viral proteins modulate and disrupt endothelial function at different stages of the infectious cycle. Importantly, cellular damage can be induced by secreted viral proteins, hence, uninfected endothelial cells can also be affected.

## Concluding Remarks

We have reviewed endothelial cell-virus interactions in emerging viral infections. We aimed to illustrate the diversity of such responses and identify common features that will bring us closer to understanding the role of endothelial cells in viral disease. This is important. First, because many pathogens that pose a threat to global human and animal health infect endothelium; second, because conditions characterised by endothelial dysfunction is associated with severe outcomes of infection with SARS-CoV-2; and third, because dysregulation of vascular function confers disease in many life-threatening viral infections.

All possible viral entry ports are in close contact with endothelial cells, and the diversity of endothelial phenotypes probably contributes to guiding viral infection and pathology. In addition, the switch from a non-pathogenic to a virulent phenotype that causes disseminated infection and generalised disease is associated with a gain in endotheliotropism, at least for some viruses. This endothelial tropism is determined by both virus and host factors that regulate virus attachment, fusion, and replication. The viral attachment has a prominent role in species- and tissue-tropism but is not sufficient for infection to occur. Rather, the viral fusion process appears to be a strong determinant of cellular tropism. Some essential features of endothelial cells that regulate such viral fusion include inefficient endosomal acidification and high levels of antiviral proteins in endosomal compartments (159, 161). Hence, an increased ability to fuse on the cellular surface or in early endosomal compartments may facilitate, but is not absolutely required, for endothelial infection. This may for example be achieved by conformational destabilisation of the fusion protein that makes it more susceptible to activation (159, 168). Much of the work that has identified specific factors regulating endothelial tropism comes from the study of orthomyxoviruses. Hence, efforts should be made to understand if these factors also determine endotheliotropism in other viruses. Studies of viral fusion are challenging in other settings than cell culture, but should be interpreted with a view to natural infections.

A systemic inflammatory response syndrome with the breakdown of vascular function characterises severe viral disease, and it has clear parallels to bacterial septic shock (89). There is a loss of control of vascular permeability, ranging from local oedema to severe hypovolemic shock, and a microvascular thrombosis that may proceed to consumptive coagulopathy. Despite the similarity of clinical signs observed in infection by different endotheliotropic viruses, the underlying disease mechanisms differ. For example, the extent of direct endothelial damage and endotheliitis vary widely, from extensive in henipavirus and SARS-CoV-2 infection (2, 3, 9) to minimal in hantavirus (11, 41) and ISAV (13) infection. Nevertheless, all these agents cause severe vascular compromise, oedema, and bleeding. This has two implications. First, mechanisms other than direct endothelial damage may cause vascular dysfunction in viral disease. Second, the immunomodulatory role of endothelium probably limits T cell killing of infected endothelial cells in some viral infections, possibly providing a niche for viral replication (192). Activation of inflammatory and antiviral pathways in endothelial cells orchestrate leukocyte recruitment to sites of infection and generate ISGs that potently inhibit viral replication. However, the same pathways also disrupt vascular function (33, 204, 234) by inducing a pro-inflammatory, pro-thrombotic vascular profile. In addition, direct interactions between viral proteins and endothelial components that regulate the vascular barrier contributes to the vascular leakage seen in viral disease (84, 92, 119, 195, 227, 228).

The biosecurity risks associated with many emerging viruses together with the invasiveness of sampling required for obtaining endothelial cells from patients, limit studies of viruses during natural human infection. However, all available model systems have significant limitations. To this end, we propose that experiments in cell and laboratory animal models, studies of animal pathogens in their natural hosts, and analysis of patient materials must be combined to fully understand viral interactions with the endothelium during infection and disease.

We hope that this article is a step toward a more generalised understanding of how endothelial cells are targeted in viral disease, either by direct infection or as bystanders. Such knowledge is likely to improve strategies for managing patients with viral infection. This is particularly important in crises like the current pandemic, where resources are exhausted by the high prevalence of infection. A better understanding of underlying mechanisms could help us evaluate the rationale of suggested strategies. One example is the stratification of risk in patients by FMD in COVID-19 (7), another the use of pleiomorphic therapies, including statins, glucocorticoids, and ACE-inhibitors, to stabilise vascular function in a non-specific manner in severe viral infection (235, 236).

## Author Contributions

JF: Concept. JF and RE: First draft. JF, RE, KF, and GH: Developing manuscript into final version. All authors contributed to the article and approved the submitted version.

## Conflict of Interest

KF was employed by the company AquaMed Consulting AS. The remaining authors declare that the research was conducted in the absence of any commercial or financial relationships that could be construed as a potential conflict of interest.

## References

[1] R&D blueprints. Key Actions by Disease. Oslo: World Health Organisation (2020).

[2] Varga Z Flammer AJ Steiger P Haberecker M Andermatt R Zinkernagel AS. Endothelial cell infection and endotheliitis in COVID-19. Lancet. (2020) 395:1417–8. 10.1016/S0140-6736(20)30937-5 32325026 PMC7172722

[3] Ackermann M Verleden SE Kuehnel M Haverich A Welte T Laenger F. Pulmonary vascular endothelialitis, thrombosis, and angiogenesis in Covid-19. N Engl J Med. (2020) 383:120–8. 10.1056/NEJMoa2015432 32437596 PMC7412750

[4] Colmenero I Santonja C Alonso-Riano M Noguera-Morel L Hernandez-Martin A Andina D. SARS-CoV-2 endothelial infection causes COVID-19 chilblains: histopathological, immunohistochemical and ultrastructural study of seven paediatric cases. Br J Dermatol. (2020) 183:729–37. 10.1111/bjd.19327 32562567 PMC7323219

[5] Monteil V Kwon H Prado P Hagelkruys A Wimmer RA Stahl M. Inhibition of SARS-CoV-2 infections in engineered human tissues using clinical-grade soluble human ACE2. Cell. (2020) 181:905–13 e7. 10.1016/j.cell.2020.04.004 32333836 PMC7181998

[6] Wazny V Siau A Wu KX Cheung C. Vascular underpinning of COVID-19. Open Biol. (2020) 10:200208. 10.1098/rsob.200208 32847471 PMC7479931

[7] Evans PC Ed Rainger G Mason JC Guzik TJ Osto E Stamataki Z. Endothelial dysfunction in COVID-19: a position paper of the ESC Working Group for Atherosclerosis and Vascular Biology, and the ESC Council of Basic Cardiovascular Science. Cardiovasc Res. (2020) 116:2177–84. 10.1093/cvr/cvaa230 32750108 PMC7454368

[8] Teuwen LA Geldhof V Pasut A Carmeliet P. COVID-19: the vasculature unleashed. Nat Rev Immunol. (2020) 20:389–91. 10.1038/s41577-020-0343-0 32439870 PMC7240244

[9] Wong KT Shieh WJ Kumar S Norain K Abdullah W Guarner J. Nipah virus infection - Pathology and pathogenesis of an emerging paramyxoviral zoonosis. Am J Pathol. (2002) 161:2153–67. 10.1016/S0002-9440(10)64493-8 12466131 PMC1850894

[10] Geisbert TW Daddario-DiCaprio KM Hickey AC Smith MA Chan YP Wang LF. Development of an acute and highly pathogenic nonhuman primate model of Nipah virus infection. PLoS ONE. (2010) 5:e10690. 10.1371/journal.pone.0010690 20502528 PMC2872660

[11] Zaki SR Greer PW Coffield LM Goldsmith CS Nolte KB Foucar K. Hantavirus pulmonary syndrome. Pathogenesis of an emerging infectious disease. Am J Pathol. (1995) 146:552–79. 7887439 PMC1869168

[12] Kuiken T van den Brand J van Riel D Pantin-Jackwood M Swayne DE. Comparative pathology of select agent influenza a virus infections. Vet Pathol. (2010) 47:893–914. 10.1177/0300985810378651 20682805

[13] Aamelfot M Dale OB Weli SC Koppang EO Falk K. Expression of the infectious salmon anemia virus receptor on atlantic salmon endothelial cells correlates with the cell tropism of the virus. J Virol. (2012) 86:10571–8. 10.1128/JVI.00047-12 22811536 PMC3457268

[14] Jessie K Fong MY Devi S Lam SK Wong KT. Localization of dengue virus in naturally infected human tissues, by immunohistochemistry and in situ hybridization. J Infect Dis. (2004) 189:1411–8. 10.1086/383043 15073678

[15] Martines RB Ng DL Greer PW Rollin PE Zaki SR. Tissue and cellular tropism, pathology and pathogenesis of Ebola and Marburg viruses. J Pathol. (2015) 235:153–74. 10.1002/path.4456 25297522

[16] Geisbert TW Young HA Jahrling PB Davis KJ Larsen T Kagan E. Pathogenesis of ebola hemorrhagic fever in primate models. Am J Pathol. (2003) 163:2371–82. 10.1016/S0002-9440(10)63592-4 14633609 PMC1892396

[17] Povoa TF Alves AM Oliveira CA Nuovo GJ Chagas VL Paes MV. The pathology of severe dengue in multiple organs of human fatal cases: histopathology, ultrastructure and virus replication. PLoS ONE. (2014) 9:e83386. 10.1371/journal.pone.0083386 24736395 PMC3987999

[18] Jin Y Ji W Yang H Chen S Zhang W Duan G. Endothelial activation and dysfunction in COVID-19: from basic mechanisms to potential therapeutic approaches. Signal Transduct Target Ther. (2020) 5:293. 10.1038/s41392-020-00454-7 33361764 PMC7758411

[19] Davidson AM Wysocki J Batlle D. Interaction of SARS-CoV-2 and other coronavirus with ACE (Angiotensin-Converting Enzyme)-2 as their main receptor: therapeutic implications. Hypertension. (2020) 76:1339–49. 10.1161/HYPERTENSIONAHA.120.15256 32851855 PMC7480804

[20] Sender R Fuchs S Milo R. Revised estimates for the number of human and bacteria cells in the body. PLoS Biol. (2016) 14:e1002533. 10.1371/journal.pbio.1002533 27541692 PMC4991899

[21] Aird WC. Phenotypic heterogeneity of the endothelium: II. Representative vascular beds. Circ Res. (2007) 100:174–90. 10.1161/01.RES.0000255690.03436.ae 17272819

[22] Jakab M Augustin HG. Understanding angiodiversity: insights from single cell biology. Development. (2020) 147:1–13. 10.1242/dev.146621 32792338

[23] Meertens L Carnec X Lecoin MP Ramdasi R Guivel-Benhassine F Lew E Lemke G Schwartz O Amara A. The TIM and TAM families of phosphatidylserine receptors mediate dengue virus entry. Cell Host Microbe. (2012) 12:544–57. 10.1016/j.chom.2012.08.009 23084921 PMC3572209

[24] Seternes T Sorensen K Smedsrod B. Scavenger endothelial cells of vertebrates: a nonperipheral leukocyte system for high-capacity elimination of waste macromolecules. Proc Natl Acad Sci USA. (2002) 99:7594–7. 10.1073/pnas.102173299 12032328 PMC124295

[25] Aird WC. Mechanisms of endothelial cell heterogeneity in health and disease. Circ Res. (2006) 98:159–62. 10.1161/01.RES.0000204553.32549.a7 16456105

[26] Lawson ND Scheer N Pham VN Kim CH Chitnis AB Campos-Ortega JA. Notch signaling is required for arterial-venous differentiation during embryonic vascular development. Development. (2001) 128:3675–83. 11585794 10.1242/dev.128.19.3675

[27] Aitsebaomo J Portbury AL Schisler JC Patterson C. Brothers and sisters: molecular insights into arterial-venous heterogeneity. Circ Res. (2008) 103:929–39. 10.1161/CIRCRESAHA.108.184937 18948631 PMC2760069

[28] Aird WC. Endothelial cell heterogeneity. Cold Spring Harb Perspect Med. (2012) 2:a006429. 10.1101/cshperspect.a006429 22315715 PMC3253027

[29] Benedito R Roca C Sorensen I Adams S Gossler A Fruttiger M. The notch ligands Dll4 and Jagged1 have opposing effects on angiogenesis. Cell. (2009) 137:1124–35. 10.1016/j.cell.2009.03.025 19524514

[30] Aird WC. Phenotypic heterogeneity of the endothelium: I. Structure, function, and mechanisms. Circ Res. (2007) 100:158–73. 10.1161/01.RES.0000255691.76142.4a 17272818

[31] Regan ER Aird WC. Dynamical systems approach to endothelial heterogeneity. Circ Res. (2012) 111:110–30. 10.1161/CIRCRESAHA.111.261701 22723222 PMC3400545

[32] Pober JS Sessa WC. Evolving functions of endothelial cells in inflammation. Nat Rev Immunol. (2007) 7:803–15. 10.1038/nri2171 17893694

[33] Liao JK. Linking endothelial dysfunction with endothelial cell activation. J Clin Invest. (2013) 123:540–1. 10.1172/JCI66843 23485580 PMC3561809

[34] Celermajer DS Sorensen KE Gooch VM Spiegelhalter DJ Miller OI Sullivan ID. Non-invasive detection of endothelial dysfunction in children and adults at risk of atherosclerosis. Lancet. (1992) 340:1111–5. 10.1016/0140-6736(92)93147-F 1359209

[35] Walls AC Park YJ Tortorici MA Wall A McGuire AT Veesler D. Structure, function, and antigenicity of the SARS-CoV-2 spike glycoprotein. Cell. (2020) 181:281–92.e6. 10.1016/j.cell.2020.02.058 32155444 PMC7102599

[36] Hoffmann M Kleine-Weber H Schroeder S Kruger N Herrler T Erichsen S. SARS-CoV-2 cell entry depends on ACE2 and TMPRSS2 and is blocked by a clinically proven protease inhibitor. Cell. (2020) 181:271–80 e8. 10.1016/j.cell.2020.02.052 32142651 PMC7102627

[37] Yan R Zhang Y Li Y Xia L Guo Y Zhou Q. Structural basis for the recognition of SARS-CoV-2 by full-length human ACE2. Science. (2020) 367:1444–8. 10.1126/science.abb2762 32132184 PMC7164635

[38] Paz Ocaranza M Riquelme JA Garcia L Jalil JE Chiong M Santos RAS. Counter-regulatory renin-angiotensin system in cardiovascular disease. Nat Rev Cardiol. (2020) 17:116–29. 10.1038/s41569-019-0244-8 31427727 PMC7097090

[39] Price LC McCabe C Garfield B Wort SJ. Thrombosis and COVID-19 pneumonia: the clot thickens! *Eur Respir J*. (2020) 56:2001608. 10.1183/13993003.01608-2020 32554532 PMC7301830

[40] Levi M van der Poll T. Coagulation and sepsis. Thromb Res. (2017) 149:38–44. 10.1016/j.thromres.2016.11.007 27886531

[41] Mackow ER Gorbunova EE Gavrilovskaya IN. Endothelial cell dysfunction in viral hemorrhage and edema. Front Microbiol. (2014) 5:733. 10.3389/fmicb.2014.00733 25601858 PMC4283606

[42] Weinbaum S Tarbell JM Damiano ER. The structure and function of the endothelial glycocalyx layer. Annu Rev Biomed Eng. (2007) 9:121–67. 10.1146/annurev.bioeng.9.060906.151959 17373886

[43] Wasik BR Barnard KN Parrish CR. Effects of sialic acid modifications on virus binding and infection. Trends Microbiol. (2016) 24:991–1001. 10.1016/j.tim.2016.07.005 27491885 PMC5123965

[44] Betteridge KB Arkill KP Neal CR Harper SJ Foster RR Satchell SC. Sialic acids regulate microvessel permeability, revealed by novel *in vivo* studies of endothelial glycocalyx structure and function. J Physiol. (2017) 595:5015–5035. 10.1113/JP274167 28524373 PMC5538239

[45] Dejana E. Endothelial cell-cell junctions: happy together. Nat Rev Mol Cell Biol. (2004) 5:261–70. 10.1038/nrm1357 15071551

[46] Duong CN Vestweber D. Mechanisms ensuring endothelial junction integrity beyond VE-cadherin. Front Physiol. (2020) 11:519. 10.3389/fphys.2020.00519 32670077 PMC7326147

[47] Giannotta M Trani M Dejana E. VE-cadherin and endothelial adherens junctions: active guardians of vascular integrity. Dev Cell. (2013) 26:441–54. 10.1016/j.devcel.2013.08.020 24044891

[48] Frye M Dierkes M Kuppers V Vockel M Tomm J Zeuschner D. Interfering with VE-PTP stabilizes endothelial junctions *in vivo* via Tie-2 in the absence of VE-cadherin. J Exp Med. (2015) 212:2267–87. 10.1084/jem.20150718 26642851 PMC4689167

[49] Ley K Laudanna C Cybulsky MI Nourshargh S. Getting to the site of inflammation: the leukocyte adhesion cascade updated. Nat Rev Immunol. (2007) 7:678–89. 10.1038/nri2156 17717539

[50] Lawrence MB Kansas GS Kunkel EJ Ley K. Threshold levels of fluid shear promote leukocyte adhesion through selectins (CD62L,P,E). J Cell Biol. (1997) 136:717–27. 10.1083/jcb.136.3.717 9024700 PMC2134292

[51] Frenette PS Mayadas TN Rayburn H Hynes RO Wagner DD. Susceptibility to infection and altered hematopoiesis in mice deficient in both P- and E-selectins. Cell. (1996) 84:563–74. 10.1016/S0092-8674(00)81032-6 8598043

[52] Pruenster M Mudde L Bombosi P Dimitrova S Zsak M Middleton J. The Duffy antigen receptor for chemokines transports chemokines and supports their promigratory activity. Nat Immunol. (2009) 10:101–8. 10.1038/ni.1675 19060902 PMC3205989

[53] Pinsky DJ Naka Y Liao H Oz MC Wagner DD Mayadas TN. Hypoxia-induced exocytosis of endothelial cell Weibel-Palade bodies. A mechanism for rapid neutrophil recruitment after cardiac preservation. J Clin Invest. (1996) 97:493–500. 10.1172/JCI118440 8567972 PMC507042

[54] Fiedler U Scharpfenecker M Koidl S Hegen A Grunow V Schmidt JM. The Tie-2 ligand angiopoietin-2 is stored in and rapidly released upon stimulation from endothelial cell Weibel-Palade bodies. Blood. (2004) 103:4150–6. 10.1182/blood-2003-10-3685 14976056

[55] Eppihimer MJ Wolitzky B Anderson DC Labow MA Granger DN. Heterogeneity of expression of E- and P-selectins *in vivo*. Circ Res. (1996) 79:560–9. 10.1161/01.RES.79.3.560 8781489

[56] Utgaard JO Jahnsen FL Bakka A Brandtzaeg P Haraldsen G. Rapid secretion of prestored interleukin 8 from Weibel-Palade bodies of microvascular endothelial cells. J Exp Med. (1998) 188:1751–6. 10.1084/jem.188.9.1751 9802986 PMC2212514

[57] Hakanpaa L Kiss EA Jacquemet G Miinalainen I Lerche M Guzman C. Targeting beta1-integrin inhibits vascular leakage in endotoxemia. Proc Natl Acad Sci USA. (2018) 115:E6467–76. 10.1073/pnas.1722317115 29941602 PMC6048499

[58] Thomas M Augustin HG. The role of the Angiopoietins in vascular morphogenesis. Angiogenesis. (2009) 12:125–37. 10.1007/s10456-009-9147-3 19449109

[59] Coultas L Chawengsaksophak K Rossant J. Endothelial cells and VEGF in vascular development. Nature. (2005) 438:937–45. 10.1038/nature04479 16355211

[60] Murakami M Signaling required for blood vessel maintenance: molecular basis and pathological manifestations. Int J Vasc Med. (2012) 2012:293641. 10.1155/2012/293641 22187650 PMC3236483

[61] Lampugnani MG Orsenigo F Gagliani MC Tacchetti C Dejana E. Vascular endothelial cadherin controls VEGFR-2 internalization and signaling from intracellular compartments. J Cell Biol. (2006) 174:593–604. 10.1083/jcb.200602080 16893970 PMC2064264

[62] Murakami M Simons M. Regulation of vascular integrity. J Mol Med. (2009) 87:571–82. 10.1007/s00109-009-0463-2 19337719 PMC2866175

[63] Tzima E Irani-Tehrani M Kiosses WB Dejana E Schultz DA Engelhardt B. A mechanosensory complex that mediates the endothelial cell response to fluid shear stress. Nature. (2005) 437:426–31. 10.1038/nature03952 16163360

[64] Fukuhara S Sako K Minami T Noda K Kim HZ Kodama T. Differential function of Tie2 at cell-cell contacts and cell-substratum contacts regulated by angiopoietin-1. Nat Cell Biol. (2008) 10:513–26. 10.1038/ncb1714 18425120

[65] Gavard J Patel V Gutkind JS. Angiopoietin-1 prevents VEGF-induced endothelial permeability by sequestering Src through mDia. Dev Cell. (2008) 14:25–36. 10.1016/j.devcel.2007.10.019 18194650

[66] Cartier A Leigh T Liu CH Hla T. Endothelial sphingosine 1-phosphate receptors promote vascular normalization and antitumor therapy. Proc Natl Acad Sci USA. (2020) 117:3157–66. 10.1073/pnas.1906246117 31988136 PMC7022165

[67] Bjarnegard M Enge M Norlin J Gustafsdottir S Fredriksson S Abramsson A. Endothelium-specific ablation of PDGFB leads to pericyte loss and glomerular, cardiac and placental abnormalities. Development. (2004) 131:1847–57. 10.1242/dev.01080 15084468

[68] Okamoto T Suzuki K. The role of gap junction-mediated endothelial cell-cell interaction in the crosstalk between inflammation and blood coagulation. Int J Mol Sci. (2017) 18:2254. 10.3390/ijms18112254 29077057 PMC5713224

[69] Kovacs-Oller T Ivanova E Bianchimano P Sagdullaev BT. The pericyte connectome: spatial precision of neurovascular coupling is driven by selective connectivity maps of pericytes and endothelial cells and is disrupted in diabetes. Cell Discov. (2020) 6:39. 10.1038/s41421-020-0180-0 32566247 PMC7296038

[70] Gralinski LE Baric RS. Molecular pathology of emerging coronavirus infections. J Pathol. (2015) 235:185–95. 10.1002/path.4454 25270030 PMC4267971

[71] Ramsey C Kumar A. H1N1: viral pneumonia as a cause of acute respiratory distress syndrome. Curr Opin Crit Care. (2011) 17:64–71. 10.1097/MCC.0b013e3283427259 21157318

[72] Shah RD Wunderink RG. Viral pneumonia and acute respiratory distress syndrome. Clin Chest Med. (2017) 38:113–25. 10.1016/j.ccm.2016.11.013 28159154 PMC7131505

[73] Bellani G Laffey JG Pham T Fan E Brochard L Esteban A. Patterns of care, and mortality for patients with acute respiratory distress syndrome in intensive care units in 50 countries. JAMA. (2016) 315:788–800. 10.1001/jama.2016.0291 26903337

[74] Ashbaugh DG Bigelow DB Petty TL Levine BE. Acute respiratory distress in adults. Lancet. (1967) 2:319–23. 10.1016/S0140-6736(67)90168-7 4143721

[75] Thompson BT Chambers RC Liu KD. Acute respiratory distress syndrome. N Engl J Med. (2017) 377:562–72. 10.1056/NEJMra1608077 28792873

[76] Short KR Kroeze E Fouchier RAM Kuiken T. Pathogenesis of influenza-induced acute respiratory distress syndrome. Lancet Infect Dis. (2014) 14:57–69. 10.1016/S1473-3099(13)70286-X 24239327

[77] Katzenstein AL Bloor CM Leibow AA. Diffuse alveolar damage–the role of oxygen, shock, and related factors. A review. Am J Pathol. (1976) 85:209–28. 788524 PMC2032554

[78] Shieh WJ Blau DM Denison AM Deleon-Carnes M Adem P Bhatnagar J. 2009 pandemic influenza A (H1N1): pathology and pathogenesis of 100 fatal cases in the United States. Am J Pathol. (2010) 177:166–75. 10.2353/ajpath.2010.100115 20508031 PMC2893660

[79] Hendrickson CM Matthay MA. Viral pathogens and acute lung injury: investigations inspired by the SARS epidemic and the 2009 H1N1 influenza pandemic. Semin Respir Crit Care Med. (2013) 34:475–86. 10.1055/s-0033-1351122 23934716 PMC4045622

[80] Itoh Y Shinya K Kiso M Watanabe T Sakoda Y Hatta M. *In vitro* and *in vivo* characterization of new swine-origin H1N1 influenza viruses. Nature. (2009) 460:1021–5. 10.1038/nature08260 19672242 PMC2748827

[81] To KK Hung IF Li IW Lee KL Koo CK Yan WW. Delayed clearance of viral load and marked cytokine activation in severe cases of pandemic H1N1 2009 influenza virus infection. Clin Infect Dis. (2010) 50:850–9. 10.1086/650581 20136415 PMC7107930

[82] Peiris JS Cheung CY Leung CY Nicholls JM. Innate immune responses to influenza A H5N1: friend or foe? Trends Immunol. (2009) 30:574–84. 10.1016/j.it.2009.09.004 19864182 PMC5068224

[83] Teijaro JR Walsh KB Cahalan S Fremgen DM Roberts E Scott F. Endothelial cells are central orchestrators of cytokine amplification during influenza virus infection. Cell. (2011) 146:980–91. 10.1016/j.cell.2011.08.015 21925319 PMC3176439

[84] Gorbunova EE Simons MJ Gavrilovskaya IN Mackow ER. The andes virus nucleocapsid protein directs basal endothelial cell permeability by activating RhoA. mBio. (2016) 7:1–12. 10.1128/mBio.01747-16 27795403 PMC5080385

[85] Hajra A Mathai SV Ball S Bandyopadhyay D Veyseh M Chakraborty S. Management of thrombotic complications in COVID-19: an update. Drugs. (2020) 80:1553–62. 10.1007/s40265-020-01377-x 32803670 PMC7429134

[86] Bompard F Monnier H Saab I Tordjman M Abdoul H Fournier L. Pulmonary embolism in patients with COVID-19 pneumonia. Eur Respir J (2020) 56:2001365. 10.1183/13993003.01365-2020 32398297 PMC7236820

[87] Llitjos JF Leclerc M Chochois C Monsallier JM Ramakers M Auvray M. High incidence of venous thromboembolic events in anticoagulated severe COVID-19 patients. J Thromb Haemost. (2020) 18:1743–46. 10.1111/jth.14869 32320517 PMC7264774

[88] Kaplan D Casper TC Elliott CG Men S Pendleton RC Kraiss LW. VTE incidence and risk factors in patients with severe sepsis and septic shock. Chest. (2015) 148:1224–30. 10.1378/chest.15-0287 26111103 PMC4631038

[89] Li H Liu L Zhang D Xu J Dai H Tang N. SARS-CoV-2 and viral sepsis: observations and hypotheses. Lancet. (2020) 395:1517–20. 10.1016/S0140-6736(20)30920-X 32311318 PMC7164875

[90] Kox M Waalders NJB Kooistra EJ Gerretsen J Pickkers P. Cytokine levels in critically ill patients with COVID-19 and other conditions. JAMA. (2020) 324:1565–7. 10.1001/jama.2020.17052 32880615 PMC7489366

[91] Halstead SB. Pathogenesis of dengue: dawn of a new era. F1000Res. (2015) 4:F1000 Faculty Rev-1353. 10.12688/f1000research.7024.1 26918141 PMC4754012

[92] Modhiran N Watterson D Muller DA Panetta AK Sester DP Liu L. Dengue virus NS1 protein activates cells via Toll-like receptor 4 and disrupts endothelial cell monolayer integrity. Sci Transl Med. (2015) 7:304ra142. 10.1126/scitranslmed.aaa3863 26355031

[93] Aird WC. The role of the endothelium in severe sepsis and multiple organ dysfunction syndrome. Blood. (2003) 101:3765–77. 10.1182/blood-2002-06-1887 12543869

[94] Ouldali N Pouletty M Mariani P Beyler C Blachier A Bonacorsi S. Emergence of Kawasaki disease related to SARS-CoV-2 infection in an epicentre of the French COVID-19 epidemic: a time-series analysis. Lancet Child Adolesc Health. (2020) 4:662–8. 10.1016/S2352-4642(20)30175-9 32622376 PMC7332278

[95] Toubiana J Poirault C Corsia A Bajolle F Fourgeaud J Angoulvant F. Kawasaki-like multisystem inflammatory syndrome in children during the covid-19 pandemic in Paris, France: prospective observational study. BMJ. (2020) 369:m2094. 10.1136/bmj.m2094 32493739 PMC7500538

[96] Richard AS Shim BS Kwon YC Zhang R Otsuka Y Schmitt K. AXL-dependent infection of human fetal endothelial cells distinguishes Zika virus from other pathogenic flaviviruses. Proc Natl Acad Sci USA. (2017) 114:2024–9. 10.1073/pnas.1620558114 28167751 PMC5338370

[97] Hussmann KL Samuel MA Kim KS Diamond MS Fredericksen BL. Differential replication of pathogenic and nonpathogenic strains of West Nile virus within astrocytes. J Virol. (2013) 87:2814–22. 10.1128/JVI.02577-12 23269784 PMC3571364

[98] Richard M Fouchier R Monne I Kuiken T. Mechanisms and risk factors for mutation from low to highly pathogenic avian influenza virus. Eur Food Saf Author. (2017) 14:1287E. 10.2903/sp.efsa.2017.EN-1287

[99] Feldmann A Schafer MK Garten W Klenk HD. Targeted infection of endothelial cells by avian influenza virus A/FPV/Rostock/34 (H7N1) in chicken embryos. J Virol. (2000) 74:8018–27. 10.1128/JVI.74.17.8018-8027.2000 10933711 PMC112334

[100] Wibawa H Bingham J Nuradji H Lowther S Payne J Harper J. The pathobiology of two Indonesian H5N1 avian influenza viruses representing different clade 2.1 sublineages in chickens and ducks. Comp Immunol Microbiol Infect Dis. (2013) 36:175–91. 10.1016/j.cimid.2012.12.001 23290928

[101] Ocana-Macchi M Bel M Guzylack-Piriou L Ruggli N Liniger M McCullough KC. Hemagglutinin-dependent tropism of H5N1 avian influenza virus for human endothelial cells. J Virol. (2009) 83:12947–55. 10.1128/JVI.00468-09 19812146 PMC2786827

[102] Short KR Veldhuis Kroeze EJ Reperant LA Richard M Kuiken T. Influenza virus and endothelial cells: a species specific relationship. Front Microbiol. (2014) 5:653. 10.3389/fmicb.2014.00653 25520707 PMC4251441

[103] Tundup S Kandasamy M Perez JT Mena N Steel J Nagy T. Endothelial cell tropism is a determinant of H5N1 pathogenesis in mammalian species. PLoS Pathog. (2017) 13:e1006270. 10.1371/journal.ppat.1006270 28282445 PMC5362246

[104] Steuerman Y Cohen M Peshes-Yaloz N Valadarsky L Cohn O David E. Dissection of influenza infection *in vivo* by single-cell RNA sequencing. Cell Syst. (2018) 6:679–91 e4. 10.1016/j.cels.2018.05.008 29886109 PMC7185763

[105] Aamelfot M Christiansen DH Dale OB McBeath A Benestad SL Falk K. Localised infection of atlantic salmon epithelial cells by HPR0 infectious salmon anaemia virus. PLoS ONE. (2016) 11:e0151723. 10.1371/journal.pone.0151723 26999815 PMC4801213

[106] Conforti G Dominguezjimenez C Zanetti A Gimbrone MA Cremona O Marchisio PC. Human endothelial-cells express integrin receptors on the luminal aspect of their membrane. Blood. (1992) 80:437–46. 10.1182/blood.V80.2.437.bloodjournal802437 1627801

[107] Stencel-Baerenwald JE Reiss K Reiter DM Stehle T Dermody TS. The sweet spot: defining virus-sialic acid interactions. Nat Rev Microbiol. (2014) 12:739–49. 10.1038/nrmicro3346 25263223 PMC4791167

[108] Long JS Mistry B Haslam SM Barclay WS. Host and viral determinants of influenza A virus species specificity. Nat Rev Microbiol. (2018) 17:67–81. 10.1038/s41579-018-0115-z 30487536

[109] Dou D Revol R Ostbye H Wang H Daniels R. Influenza A virus cell entry, replication, virion assembly and movement. Front Immunol. (2018) 9:1581. 10.3389/fimmu.2018.01581 30079062 PMC6062596

[110] Hellebo A Vilas U Falk K Vlasak R. Infectious salmon anemia virus specifically binds to and hydrolyzes 4-O-acetylated sialic acids. J Virol. (2004) 78:3055–62. 10.1128/JVI.78.6.3055-3062.2004 14990724 PMC353765

[111] Zeng H Pappas C Belser JA Houser KV Zhong W Wadford DA. Human pulmonary microvascular endothelial cells support productive replication of highly pathogenic avian influenza viruses: possible involvement in the pathogenesis of human H5N1 virus infection. J Virol. (2012) 86:667–78. 10.1128/JVI.06348-11 22072765 PMC3255832

[112] Ayala-Nunez NV Gaudin R. A viral journey to the brain: current considerations and future developments. PLoS Pathog. (2020) 16:e1008434. 10.1371/journal.ppat.1008434 32437459 PMC7241694

[113] Mathieu C Pohl C Szecsi J Trajkovic-Bodennec S Devergnas S Raoul H. Nipah virus uses leukocytes for efficient dissemination within a host. J Virol. (2011) 85:7863–71. 10.1128/JVI.00549-11 21593145 PMC3147937

[114] Koren CWR Nylund A. Morphology and morphogenesis of infectious salmon anaemia virus replicating in the endothelium of Atlantic salmon Salmo salar. Dise Aquat Org. (1997) 29:99–109. 10.3354/dao029099

[115] Klenk HD. Infection of the endothelium by influenza viruses. Thromb Haemost. (2005) 94:262–5. 10.1160/TH05-04-0264 16113814

[116] Hasebe R Suzuki T Makino Y Igarashi M Yamanouchi S Maeda A. Transcellular transport of West Nile virus-like particles across human endothelial cells depends on residues 156 and 159 of envelope protein. BMC Microbiol. (2010) 10:165. 10.1186/1471-2180-10-165 20529314 PMC2889955

[117] Chiu CF Chu LW Liao IC Simanjuntak Y Lin YL Juan CC. The mechanism of the zika virus crossing the placental barrier and the blood-brain barrier. Front Microbiol. (2020) 11:214. 10.3389/fmicb.2020.00214 32153526 PMC7044130

[118] Wang T Town T Alexopoulou L Anderson JF Fikrig E Flavell RA. Toll-like receptor 3 mediates West Nile virus entry into the brain causing lethal encephalitis. Nat Med. (2004) 10:1366–73. 10.1038/nm1140 15558055

[119] Puerta-Guardo H Glasner DR Espinosa DA Biering SB Patana M Ratnasiri K. Flavivirus NS1 triggers tissue-specific vascular endothelial dysfunction reflecting disease tropism. Cell Rep. (2019) 26:1598–613 e8. 10.1016/j.celrep.2019.01.036 30726741 PMC6934102

[120] Navaratnarajah CK Generous AR Yousaf I Cattaneo R. Receptor-mediated cell entry of paramyxoviruses: Mechanisms, and consequences for tropism and pathogenesis. J Biol Chem. (2020) 295:2771–86. 10.1074/jbc.REV119.009961 31949044 PMC7049954

[121] Morizono K Chen IS. Receptors and tropisms of envelope viruses. Curr Opin Virol. (2011) 1:13–8. 10.1016/j.coviro.2011.05.001 21804908 PMC3144558

[122] Olofsson S Bergstrom T. Glycoconjugate glycans as viral receptors. Ann Med. (2005) 37:154–72. 10.1080/07853890510007340 16019714

[123] van Riel D Munster VJ de Wit E Rimmelzwaan GF Fouchier RA Osterhaus AD. Human and avian influenza viruses target different cells in the lower respiratory tract of humans and other mammals. Am J Pathol. (2007) 171:1215–23. 10.2353/ajpath.2007.070248 17717141 PMC1988871

[124] Ma D Chen CB Jhanji V Xu C Yuan XL Liang JJ. Expression of SARS-CoV-2 receptor ACE2 and TMPRSS2 in human primary conjunctival and pterygium cell lines and in mouse cornea. Eye. (2020) 34:1212–9. 10.1038/s41433-020-0939-4 32382146 PMC7205026

[125] Lukassen S Chua RL Trefzer T Kahn NC Schneider MA Muley T. SARS-CoV-2 receptor ACE2 and TMPRSS2 are primarily expressed in bronchial transient secretory cells. EMBO J. (2020) 39:e105114. 10.15252/embj.2020105114 32246845 PMC7232010

[126] Hamming I Timens W Bulthuis ML Lely AT Navis G van Goor H. Tissue distribution of ACE2 protein, the functional receptor for SARS coronavirus. A first step in understanding SARS pathogenesis. J Pathol. (2004) 203:631–7. 10.1002/path.1570 15141377 PMC7167720

[127] Li F. Receptor recognition mechanisms of coronaviruses: a decade of structural studies. J Virol. (2015) 89:1954–64. 10.1128/JVI.02615-14 25428871 PMC4338876

[128] Yang J Petitjean SJL Koehler M Zhang Q Dumitru AC Chen W. Molecular interaction and inhibition of SARS-CoV-2 binding to the ACE2 receptor. Nat Commun. (2020) 11:4541. 10.1038/s41467-020-18319-6 32917884 PMC7486399

[129] Aamelfot M Dale OB McBeath A Falk K. Host tropism of infectious salmon anaemia virus in marine and freshwater fish species. J Fish Dis. (2015) 38:687–94. 10.1111/jfd.12284 25048819

[130] Wang K Chen W Zhang Z Deng Y Lian JQ Du P. CD147-spike protein is a novel route for SARS-CoV-2 infection to host cells. Signal Transduct Target Ther. (2020) 5:283. 10.1038/s41392-020-00426-x 33277466 PMC7714896

[131] Benton DJ Wrobel AG Xu P Roustan C Martin SR Rosenthal PB. Receptor binding and priming of the spike protein of SARS-CoV-2 for membrane fusion. Nature. (2020) 588:327–30. 10.1038/s41586-020-2772-0 32942285 PMC7116727

[132] Negrete OA Levroney EL Aguilar HC Bertolotti-Ciarlet A Nazarian R Tajyar S. EphrinB2 is the entry receptor for Nipah virus, an emergent deadly paramyxovirus. Nature. (2005) 436:401–5. 10.1038/nature03838 16007075

[133] Mittler E Dieterle ME Kleinfelter LM Slough MM Chandran K Jangra RK. Hantavirus entry: Perspectives and recent advances. Adv Virus Res. (2019) 104:185–224. 10.1016/bs.aivir.2019.07.002 31439149 PMC6881143

[134] Chutinimitkul S van Riel D Munster VJ van den Brand JM Rimmelzwaan GF Kuiken T. *In vitro* assessment of attachment pattern and replication efficiency of H5N1 influenza A viruses with altered receptor specificity. J Virol. (2010) 84:6825–33. 10.1128/JVI.02737-09 20392847 PMC2903244

[135] Matrosovich M Tuzikov A Bovin N Gambaryan A Klimov A Castrucci MR. Early alterations of the receptor-binding properties of H1, H2, and H3 avian influenza virus hemagglutinins after their introduction into mammals. J Virol. (2000) 74:8502–12. 10.1128/JVI.74.18.8502-8512.2000 10954551 PMC116362

[136] Cook JD Sultana A Lee JE. Structure of the infectious salmon anemia virus receptor complex illustrates a unique binding strategy for attachment. Proc Natl Acad Sci USA. (2017) 114:E2929–36. 10.1073/pnas.1617993114 28320973 PMC5389325

[137] Aamelfot M Dale OB Weli SC Koppang EO Falk K. The in situ distribution of glycoprotein-bound 4-O-Acetylated sialic acids in vertebrates. Glycoconj J. (2014) 31:327–35. 10.1007/s10719-014-9529-7 24833039 PMC7088174

[138] McBeath A Fourrier M Munro E Falk K Snow M. Presence of a full-length highly polymorphic region (HPR) in the ISAV haemagglutinin-esterase does not affect the primary functions of receptor binding and esterase activity. Arch Virol. (2011) 156:2285–9. 10.1007/s00705-011-1106-9 21935625

[139] Ramirez-Ortiz ZG Pendergraft WF III Prasad A Byrne MH Iram T Blanchette CJ. The scavenger receptor SCARF1 mediates the clearance of apoptotic cells and prevents autoimmunity. Nat Immunol. (2013) 14:917–26. 10.1038/ni.2670 23892722 PMC3752698

[140] Soares MM King SW Thorpe PE. Targeting inside-out phosphatidylserine as a therapeutic strategy for viral diseases. Nat Med. (2008) 14:1357–62. 10.1038/nm.1885 19029986 PMC2597367

[141] Kondratowicz AS Lennemann NJ Sinn PL Davey RA Hunt CL Moller-Tank S. T-cell immunoglobulin and mucin domain 1 (TIM-1) is a receptor for Zaire Ebolavirus and Lake Victoria Marburgvirus. Proc Natl Acad Sci USA. (2011) 108:8426–31. 10.1073/pnas.1019030108 21536871 PMC3100998

[142] Simmons G Reeves JD Grogan CC Vandenberghe LH Baribaud F Whitbeck JC. DC-SIGN and DC-SIGNR bind ebola glycoproteins and enhance infection of macrophages and endothelial cells. Virology. (2003) 305:115–23. 10.1006/viro.2002.1730 12504546

[143] Lai WK Sun PJ Zhang J Jennings A Lalor PF Hubscher S McKeating JA Adams DH. Expression of DC-SIGN and DC-SIGNR on human sinusoidal endothelium: a role for capturing hepatitis C virus particles. Am J Pathol. (2006) 169:200–8. 10.2353/ajpath.2006.051191 16816373 PMC1698775

[144] Dollery SJ. Towards understanding KSHV fusion and entry. Viruses. (2019) 11:1073. 10.3390/v11111073 31752107 PMC6893419

[145] Tassaneetrithep B Burgess TH Granelli-Piperno A Trumpfheller C Finke J Sun W. DC-SIGN (CD209) mediates dengue virus infection of human dendritic cells. J Exp Med. (2003) 197:823–9. 10.1084/jem.20021840 12682107 PMC2193896

[146] White JM Whittaker GR. Fusion of enveloped viruses in endosomes. Traffic. (2016) 17:593–614. 10.1111/tra.12389 26935856 PMC4866878

[147] Bignon EA Albornoz A Guardado-Calvo P Rey FA Tischler ND. Molecular organization and dynamics of the fusion protein Gc at the hantavirus surface. Elife. (2019) 8:e46028. 10.7554/eLife.46028 31180319 PMC6609335

[148] Kappelhoff R Puente XS Wilson CH Seth A Lopez-Otin C Overall CM. Overview of transcriptomic analysis of all human proteases, non-proteolytic homologs and inhibitors: Organ, tissue and ovarian cancer cell line expression profiling of the human protease degradome by the CLIP-CHIP DNA microarray. Biochim Biophys Acta Mol Cell Res. (2017) 1864:2210–9. 10.1016/j.bbamcr.2017.08.004 28797648

[149] Kido H Okumura Y Takahashi E Pan HY Wang S Yao D. Role of host cellular proteases in the pathogenesis of influenza and influenza-induced multiple organ failure. Biochim Biophys Acta. (2012) 1824:186–94. 10.1016/j.bbapap.2011.07.001 21801859

[150] Stieneke-Gröber A Vey M Angliker H Shaw E Thomas G Roberts C. Influenza virus hemagglutinin with multibasic cleavage site is activated by furin, a subtilisin-like endoprotease. EMBO J. (1992) 11:2407–14. 1628614 10.1002/j.1460-2075.1992.tb05305.x PMC556715

[151] Millet JK Whittaker GR. Host cell proteases: Critical determinants of coronavirus tropism and pathogenesis. Virus Res. (2015) 202:120–34. 10.1016/j.virusres.2014.11.021 25445340 PMC4465284

[152] Coutard B Valle C de Lamballerie X Canard B Seidah NG Decroly E. The spike glycoprotein of the new coronavirus 2019-nCoV contains a furin-like cleavage site absent in CoV of the same clade. Antiviral Res. (2020) 176:104742. 10.1016/j.antiviral.2020.104742 32057769 PMC7114094

[153] Shang J Wan Y Luo C Ye G Geng Q Auerbach A. Cell entry mechanisms of SARS-CoV-2. Proc Natl Acad Sci USA. (2020) 117:11727–34. 10.1073/pnas.2003138117 32376634 PMC7260975

[154] Teesalu T Sugahara KN Kotamraju VR Ruoslahti E. C-end rule peptides mediate neuropilin-1-dependent cell, vascular, and tissue penetration. Proc Natl Acad Sci USA. (2009) 106:16157–62. 10.1073/pnas.0908201106 19805273 PMC2752543

[155] Daly JL Simonetti B Klein K Chen KE Williamson MK Anton-Plagaro C. Neuropilin-1 is a host factor for SARS-CoV-2 infection. Science. (2020) 370:861–5. 10.1126/science.abd3072 33082294 PMC7612957

[156] Cantuti-Castelvetri L Ojha R Pedro LD Djannatian M Franz J Kuivanen S. Neuropilin-1 facilitates SARS-CoV-2 cell entry and infectivity. Science. (2020) 370:856–60. 10.1126/science.abd2985 33082293 PMC7857391

[157] Pager CT Dutch RE. Cathepsin L is involved in proteolytic processing of the Hendra virus fusion protein. J Virol. (2005) 79:12714–20. 10.1128/JVI.79.20.12714-12720.2005 16188974 PMC1235853

[158] Pager CT Craft WW Jr Patch J Dutch RE. A mature and fusogenic form of the Nipah virus fusion protein requires proteolytic processing by cathepsin L. Virology. (2006) 346:251–7. 10.1016/j.virol.2006.01.007 16460775 PMC7111743

[159] Hensen L Matrosovich T Roth K Klenk HD Matrosovich M. HA-dependent tropism of H5N1 and H7N9 influenza viruses to human endothelial cells is determined by reduced stability of the HA, which allows the virus to cope with inefficient endosomal acidification and constitutively expressed IFITM3. J Virol (2019) 94:e01223–19. 10.1128/JVI.01223-19 31597765 PMC6912096

[160] Sun X Zeng H Kumar A Belser JA Maines TR Tumpey TM. Constitutively expressed IFITM3 protein in human endothelial cells poses an early infection block to human influenza viruses. J Virol. (2016) 90:11157–67. 10.1128/JVI.01254-16 27707929 PMC5126373

[161] Gerlach T Hensen L Matrosovich T Bergmann J Winkler M Peteranderl C. pH optimum of hemagglutinin-mediated membrane fusion determines sensitivity of influenza a viruses to the interferon-induced antiviral state and IFITMs. J Virol. (2017) 91:e00246–17. 10.1128/JVI.00246-17 28356532 PMC5432869

[162] Brass AL Huang IC Benita Y John SP Krishnan MN Feeley EM. The IFITM proteins mediate cellular resistance to influenza A H1N1 virus, West Nile virus, and dengue virus. Cell. (2009) 139:1243–54. 10.1016/j.cell.2009.12.017 20064371 PMC2824905

[163] Xu K Chan YP Rajashankar KR Khetawat D Yan L Kolev MV. New insights into the Hendra virus attachment and entry process from structures of the virus G glycoprotein and its complex with Ephrin-B2. PLoS ONE. (2012) 7:e48742. 10.1371/journal.pone.0048742 23144952 PMC3489827

[164] Aspehaug V Mikalsen AB Snow M Biering E Villoing S. Characterization of the infectious salmon anemia virus fusion protein. J Virol. (2005) 79:12544–53. 10.1128/JVI.79.19.12544-12553.2005 16160182 PMC1211514

[165] Gagne N LeBlanc F. Overview of infectious salmon anaemia virus (ISAV) in Atlantic Canada and first report of an ISAV North American-HPR0 subtype. J Fish Dis. (2018) 41:421–30. 10.1111/jfd.12670 28782809

[166] Fourrier M Lester K Thoen E Mikalsen A Evensen O Falk K. Deletions in the highly polymorphic region (HPR) of infectious salmon anaemia virus HPR0 haemagglutinin-esterase enhance viral fusion and influence the interaction with the fusion protein. J Gen Virol. (2014) 95:1015–24. 10.1099/vir.0.061648-0 24486627

[167] Muller A Markussen T Drablos F Gjoen T Jorgensen TO Solem ST. Structural and functional analysis of the hemagglutinin-esterase of infectious salmon anaemia virus. Virus Res. (2010) 151:131–41. 10.1016/j.virusres.2010.03.020 20398710 PMC7114507

[168] Fourrier M Lester K Markussen T Falk K Secombes CJ McBeath A. Dual mutation events in the haemagglutinin-esterase and fusion protein from an infectious salmon anaemia virus HPR0 genotype promote viral fusion and activation by an ubiquitous host protease. PLoS ONE. (2015) 10:e0142020. 10.1371/journal.pone.0142020 26517828 PMC4627773

[169] Matsuyama S Nagata N Shirato K Kawase M Takeda M Taguchi F. Efficient activation of the severe acute respiratory syndrome coronavirus spike protein by the transmembrane protease TMPRSS2. J Virol. (2010) 84:12658–64. 10.1128/JVI.01542-10 20926566 PMC3004351

[170] Li M Chen L Zhang J Xiong C Li X. The SARS-CoV-2 receptor ACE2 expression of maternal-fetal interface and fetal organs by single-cell transcriptome study. PLoS ONE. (2020) 15:e0230295. 10.1371/journal.pone.0230295 32298273 PMC7161957

[171] Bertram S Heurich A Lavender H Gierer S Danisch S Perin P. Influenza and SARS-coronavirus activating proteases TMPRSS2 and HAT are expressed at multiple sites in human respiratory and gastrointestinal tracts. PLoS ONE. (2012) 7:e35876. 10.1371/journal.pone.0035876 22558251 PMC3340400

[172] Kleinfelter LM Jangra RK Jae LT Herbert AS Mittler E Stiles KM. Haploid genetic screen reveals a profound and direct dependence on cholesterol for hantavirus membrane fusion. mBio. (2015) 6:e00801. 10.1128/mBio.00801-15 26126854 PMC4488941

[173] Zaitseva E Yang ST Melikov K Pourmal S Chernomordik LV. Dengue virus ensures its fusion in late endosomes using compartment-specific lipids. PLoS Pathog. (2010) 6:e1001131. 10.1371/journal.ppat.1001131 20949067 PMC2951369

[174] Mehle A Doudna JA. Adaptive strategies of the influenza virus polymerase for replication in humans. Proc Natl Acad Sci USA. (2009) 106:21312–6. 10.1073/pnas.0911915106 19995968 PMC2789757

[175] Mehle A Dugan VG Taubenberger JK Doudna JA. Reassortment and mutation of the avian influenza virus polymerase PA subunit overcome species barriers. J Virol. (2012) 86:1750–7. 10.1128/JVI.06203-11 22090127 PMC3264373

[176] Gabriel G Klingel K Otte A Thiele S Hudjetz B Arman-Kalcek G. Differential use of importin-alpha isoforms governs cell tropism and host adaptation of influenza virus. Nat Commun. (2011) 2:156. 10.1038/ncomms1158 21245837 PMC3105303

[177] Kraus AA Raftery MJ Giese T Ulrich R Zawatzky R Hippenstiel S. Differential antiviral response of endothelial cells after infection with pathogenic and nonpathogenic hantaviruses. J Virol. (2004) 78:6143–50. 10.1128/JVI.78.12.6143-6150.2004 15163707 PMC416501

[178] McBeath A Aamelfot M Christiansen DH Matejusova I Markussen T Kaldhusdal M. Immersion challenge with low and highly virulent infectious salmon anaemia virus reveals different pathogenesis in Atlantic salmon, *Salmo salar* L. J Fish Dis. (2015) 38:3–15. 10.1111/jfd.12253 24820820

[179] McBeath AJ Ho YM Aamelfot M Hall M Christiansen DH Markussen T. Low virulent infectious salmon anaemia virus (ISAV) replicates and initiates the immune response earlier than a highly virulent virus in Atlantic salmon gills. Vet Res. (2014) 45:83. 10.1186/s13567-014-0083-x 25143055 PMC4144175

[180] Escudero-Perez B Volchkova VA Dolnik O Lawrence P Volchkov VE. Shed GP of Ebola virus triggers immune activation and increased vascular permeability. PLoS Pathog. (2014) 10:e1004509. 10.1371/journal.ppat.1004509 25412102 PMC4239094

[181] Yan N Chen ZJ. Intrinsic antiviral immunity. Nat Immunol. (2012) 13:214–22. 10.1038/ni.2229 22344284 PMC3549670

[182] Mathieu C Guillaume V Sabine A Ong KC Wong KT Legras-Lachuer C. Lethal Nipah virus infection induces rapid overexpression of CXCL10. PLoS ONE. (2012) 7:e32157. 10.1371/journal.pone.0032157 22393386 PMC3290546

[183] Schoggins JW Wilson SJ Panis M Murphy MY Jones CT Bieniasz P. A diverse range of gene products are effectors of the type I interferon antiviral response. Nature. (2011) 472:481–5. 10.1038/nature09907 21478870 PMC3409588

[184] Mussbacher M Salzmann M Brostjan C Hoesel B Schoergenhofer C Datler H. Cell type-specific roles of NF-kappaB linking inflammation and thrombosis. Front Immunol. (2019) 10:85. 10.3389/fimmu.2019.00085 30778349 PMC6369217

[185] Ablasser A Schmid-Burgk JL Hemmerling I Horvath GL Schmidt T Latz E. Cell intrinsic immunity spreads to bystander cells via the intercellular transfer of cGAMP. Nature. (2013) 503:530–4. 10.1038/nature12640 24077100 PMC4142317

[186] Spray DC Hanstein R Lopez-Quintero SV Stout RF Jr Suadicani SO Thi MM. Gap junctions and Bystander Effects: good Samaritans and executioners. Wiley Interdiscip Rev Membr Transp Signal. (2013) 2:1–15. 10.1002/wmts.72 23565352 PMC3614363

[187] Sepahi A Kraus A Casadei E Johnston CA Galindo-Villegas J Kelly C. Olfactory sensory neurons mediate ultrarapid antiviral immune responses in a TrkA-dependent manner. Proc Natl Acad Sci USA. (2019) 116:12428–36. 10.1073/pnas.1900083116 31160464 PMC6589677

[188] Feng X Li S Sun Q Zhu J Chen B Xiong M. Immune-inflammatory parameters in COVID-19 cases: a systematic review and meta-analysis. Front Med. (2020) 7:301. 10.3389/fmed.2020.00301 32582743 PMC7295898

[189] Wong KT Grosjean I Brisson C Blanquier B Fevre-Montange M Bernard A. A Golden hamster model for human acute nipah virus infection. Am J Pathol. (2003) 163:2127–37. 10.1016/S0002-9440(10)63569-9 14578210 PMC1892425

[190] Rothermel AL Wang Y Schechner J Mook-Kanamori B Aird WC Pober JS. Endothelial cells present antigens *in vivo*. BMC Immunol. (2004) 5:5. 10.1186/1471-2172-5-5 15113397 PMC394319

[191] Eppihimer MJ Gunn J Freeman GJ Greenfield EA Chernova T Erickson J. Expression and regulation of the PD-L1 immunoinhibitory molecule on microvascular endothelial cells. Microcirculation. (2002) 9:133–45. 10.1080/713774061 11932780 PMC3740166

[192] Tewalt EF Cohen JN Rouhani SJ Guidi CJ Qiao H Fahl SP. Lymphatic endothelial cells induce tolerance via PD-L1 and lack of costimulation leading to high-level PD-1 expression on CD8 T cells. Blood. (2012) 120:4772–82. 10.1182/blood-2012-04-427013 22993390 PMC3520619

[193] Mueller SN Vanguri VK Ha SJ West EE Keir ME Glickman JN. PD-L1 has distinct functions in hematopoietic and nonhematopoietic cells in regulating T cell responses during chronic infection in mice. J Clin Invest. (2010) 120:2508–15. 10.1172/JCI40040 20551512 PMC2898584

[194] Luo J Rizvi H Egger JV Preeshagul IR Wolchok JD Hellmann MD. Impact of PD-1 blockade on severity of COVID-19 in patients with lung cancers. Cancer Discov. (2020) 10:1121–8. 10.1158/2159-8290.CD-20-0596 32398243 PMC7416461

[195] Gavrilovskaya IN Gorbunova EE Mackow NA Mackow ER. Hantaviruses direct endothelial cell permeability by sensitizing cells to the vascular permeability factor VEGF, while angiopoietin 1 and sphingosine 1-phosphate inhibit hantavirus-directed permeability. J Virol. (2008) 82:5797–806. 10.1128/JVI.02397-07 18367532 PMC2395149

[196] Goodwin JE Feng Y Velazquez H Sessa WC. Endothelial glucocorticoid receptor is required for protection against sepsis. Proc Natl Acad Sci USA. (2013) 110:306–11. 10.1073/pnas.1210200110 23248291 PMC3538225

[197] WHO Rapid Evidence Appraisal for COVID-19 Therapies (REACT) Working Group„ Sterne JAC Murthy S Diaz JV Slutsky AS Villar J. Association between administration of systemic corticosteroids and mortality among critically ill patients with COVID-19: a meta-analysis. JAMA. (2020) 324:1330–41. 10.1001/jama.2020.17023 32876694 PMC7489434

[198] Tyden H Lood C Gullstrand B Nielsen CT Heegaard NHH Kahn R Jonsen A Bengtsson AA. Endothelial dysfunction is associated with activation of the type I interferon system and platelets in patients with systemic lupus erythematosus. RMD Open. (2017) 3:e000508. 10.1136/rmdopen-2017-000508 29119007 PMC5663269

[199] Jones Buie JN Oates JC. Role of interferon alpha in endothelial dysfunction: insights into endothelial nitric oxide synthase–related mechanisms. Am J Medl Sci. (2014) 348:168–175. 10.1097/MAJ.0000000000000284 24796291 PMC4526236

[200] Jia H Thelwell C Dilger P Bird C Daniels S Wadhwa M. Endothelial cell functions impaired by interferon *in vitro*: Insights into the molecular mechanism of thrombotic microangiopathy associated with interferon therapy. Thromb Res. (2018) 163:105–16. 10.1016/j.thromres.2018.01.039 29407621

[201] Dittmann M Hoffmann HH Scull MA Gilmore RH Bell KL Ciancanelli M. A serpin shapes the extracellular environment to prevent influenza A virus maturation. Cell. (2015) 160:631–43. 10.1016/j.cell.2015.01.040 25679759 PMC4328142

[202] Meigs JB O'Donnell CJ Tofler GH Benjamin EJ Fox CS Lipinska I. Hemostatic markers of endothelial dysfunction and risk of incident type 2 diabetes: the Framingham Offspring Study. Diabetes. (2006) 55:530–7. 10.2337/diabetes.55.02.06.db05-1041 16443791

[203] Ding J Song D Ye X Liu SF. A pivotal role of endothelial-specific NF-kappaB signaling in the pathogenesis of septic shock and septic vascular dysfunction. J Immunol. (2009) 183:4031–8. 10.4049/jimmunol.0900105 19692637 PMC2907363

[204] Gareus R Kotsaki E Xanthoulea S van der Made I Gijbels MJ Kardakaris R. Endothelial cell-specific NF-kappaB inhibition protects mice from atherosclerosis. Cell Metab. (2008) 8:372–83. 10.1016/j.cmet.2008.08.016 19046569

[205] Rathore AP Mantri CK Aman SA Syenina A Ooi J Jagaraj CJ. Dengue virus-elicited tryptase induces endothelial permeability and shock. J Clin Invest. (2019) 129:4180–93. 10.1172/JCI128426 31265436 PMC6763290

[206] Eliassen TM Froystad MK Dannevig BH Jankowska M Brech A Falk K. Initial events in infectious salmon anemia virus infection: evidence for the requirement of a low-pH step. J Virol. (2000) 74:218–27. 10.1128/JVI.74.1.218-227.2000 10590109 PMC111531

[207] Wong JJW Young TA Zhang J Liu S Leser GP Komives EA. Monomeric ephrinB2 binding induces allosteric changes in Nipah virus G that precede its full activation. Nat Commun. (2017) 8:781. 10.1038/s41467-017-00863-3 28974687 PMC5626764

[208] Cook JD Soto-Montoya H Korpela MK Lee JE. Electrostatic architecture of the Infectious Salmon Anemia Virus (ISAV) core fusion protein illustrates a carboxyl-carboxylate pH sensor. J Biol Chem. (2015) 290:18495–504. 10.1074/jbc.M115.644781 26082488 PMC4513110

[209] Dalrymple NA Mackow ER. Virus interactions with endothelial cell receptors: implications for viral pathogenesis. Curr Opin Virol. (2014) 7:134–40. 10.1016/j.coviro.2014.06.006 25063986 PMC4206553

[210] Saponaro F Rutigliano G Sestito S Bandini L Storti B Bizzarri R. ACE2 in the Era of SARS-CoV-2: controversies and novel perspectives. Front Mol Biosci. (2020) 7:588618. 10.3389/fmolb.2020.588618 33195436 PMC7556165

[211] Kuba K Imai Y Rao S Gao H Guo F Guan B. A crucial role of angiotensin converting enzyme 2 (ACE2) in SARS coronavirus-induced lung injury. Nat Med. (2005) 11:875–9. 10.1038/nm1267 16007097 PMC7095783

[212] Haga S Yamamoto N Nakai-Murakami C Osawa Y Tokunaga K Sata T. Modulation of TNF- -converting enzyme by the spike protein of SARS-CoV and ACE2 induces TNF- production and facilitates viral entry. Proc Natl Acad Sci USA. (2008) 105:7809–14. 10.1073/pnas.0711241105 18490652 PMC2409424

[213] Verdecchia P Cavallini C Spanevello A Angeli F. The pivotal link between ACE2 deficiency and SARS-CoV-2 infection. Eur J Intern Med. (2020) 76:14–20. 10.1016/j.ejim.2020.04.037 32336612 PMC7167588

[214] Roth L Prahst C Ruckdeschel T Savant S Westrom S Fantin A. Neuropilin-1 mediates vascular permeability independently of vascular endothelial growth factor receptor-2 activation. Sci Signal. (2016) 9:ra42. 10.1126/scisignal.aad3812 27117252

[215] Wang Y Jin G Miao H Li JY Usami S Chien S. Integrins regulate VE-cadherin and catenins: dependence of this regulation on Src, but not on Ras. Proc Natl Acad Sci USA. (2006) 103:1774–9. 10.1073/pnas.0510774103 16446427 PMC1413667

[216] Yamamoto H Ehling M Kato K Kanai K van Lessen M Frye M. Integrin beta1 controls VE-cadherin localization and blood vessel stability. Nat Commun. (2015) 6:6429. 10.1038/ncomms7429 25752958

[217] Robinson SD Reynolds LE Wyder L Hicklin DJ Hodivala-Dilke KM. Beta3-integrin regulates vascular endothelial growth factor-A-dependent permeability. Arterioscler Thromb Vasc Biol. (2004) 24:2108–14. 10.1161/01.ATV.0000143857.27408.de 15345507

[218] Raymond T Gorbunova E Gavrilovskaya IN Mackow ER. Pathogenic hantaviruses bind plexin-semaphorin-integrin domains present at the apex of inactive, bent alphavbeta3 integrin conformers. Proc Natl Acad Sci USA. (2005) 102:1163–8. 10.1073/pnas.0406743102 15657120 PMC545842

[219] Alghisi GC Ponsonnet L Ruegg C. The integrin antagonist cilengitide activates alphaVbeta3, disrupts VE-cadherin localization at cell junctions and enhances permeability in endothelial cells. PLoS ONE. (2009) 4:e4449. 10.1371/journal.pone.0004449 19212436 PMC2636874

[220] Sawamiphak S Seidel S Essmann CL Wilkinson GA Pitulescu ME Acker T. Ephrin-B2 regulates VEGFR2 function in developmental and tumour angiogenesis. Nature. (2010) 465:487–91. 10.1038/nature08995 20445540

[221] Wang Y Nakayama M Pitulescu ME Schmidt TS Bochenek ML Sakakibara A. Ephrin-B2 controls VEGF-induced angiogenesis and lymphangiogenesis. Nature. (2010) 465:483–6. 10.1038/nature09002 20445537

[222] Kim I Ryu YS Kwak HJ Ahn SY Oh JL Yancopoulos GD. EphB ligand, ephrinB2, suppresses the VEGF- and angiopoietin 1-induced Ras/mitogen-activated protein kinase pathway in venous endothelial cells. FASEB J. (2002) 16:1126–8. 10.1096/fj.01-0805fje 12039842

[223] Vreeken D Zhang H van Zonneveld AJ van Gils JM. Ephs and ephrins in adult endothelial biology. Int J Mol Sci. (2020) 21:5623. 10.3390/ijms21165623 32781521 PMC7460586

[224] Ghori A Freimann FB Nieminen-Kelha M Kremenetskaia I Gertz K Endres M. EphrinB2 activation enhances vascular repair mechanisms and reduces brain swelling after mild cerebral ischemia. Arterioscler Thromb Vasc Biol. (2017) 37:867–78. 10.1161/ATVBAHA.116.308620 28254815

[225] Frye M Stritt S Ortsater H Hernandez Vasquez M Kaakinen M Vicente A. EphrinB2-EphB4 signalling provides Rho-mediated homeostatic control of lymphatic endothelial cell junction integrity. Elife. (2020) 9:e57732. 10.7554/eLife.57732 32897857 PMC7478896

[226] Tamin A Harcourt BH Ksiazek TG Rollin PE Bellini WJ Rota PA. Functional properties of the fusion and attachment glycoproteins of Nipah virus. Virology. (2002) 296:190–200. 10.1006/viro.2002.1418 12036330

[227] van Riel D Leijten LM Kochs G Osterhaus A Kuiken T. Decrease of virus receptors during highly pathogenic H5N1 virus infection in humans and other mammals. Am J Pathol. (2013) 183:1382–9. 10.1016/j.ajpath.2013.07.004 23993779

[228] Huang IC Li W Sui J Marasco W Choe H Farzan M. Influenza A virus neuraminidase limits viral superinfection. J Virol. (2008) 82:4834–43. 10.1128/JVI.00079-08 18321971 PMC2346733

[229] Morrison TG McGinnes LW. Avian cells expressing the Newcastle disease virus hemagglutinin-neuraminidase protein are resistant to Newcastle disease virus infection. Virology. (1989) 171:10–7. 10.1016/0042-6822(89)90505-9 2545025

[230] Horga MA Gusella GL Greengard O Poltoratskaia N Porotto M Moscona A. Mechanism of interference mediated by human parainfluenza virus type 3 infection. J Virol. (2000) 74:11792–9. 10.1128/JVI.74.24.11792-11799.2000 11090179 PMC112462

[231] Tang TH Alonso S Ng LF Thein TL Pang VJ Leo YS. Increased serum hyaluronic acid and heparan sulfate in dengue fever: association with plasma leakage and disease severity. Sci Rep. (2017) 7:46191. 10.1038/srep46191 28393899 PMC5385535

[232] Puerta-Guardo H Glasner DR Harris E. Dengue virus NS1 disrupts the endothelial glycocalyx, leading to hyperpermeability. PLoS Pathog. (2016) 12:e1005738. 10.1371/journal.ppat.1005738 27416066 PMC4944995

[233] Wahl-Jensen VM Afanasieva TA Seebach J Stroher U Feldmann H Schnittler HJ. Effects of Ebola virus glycoproteins on endothelial cell activation and barrier function. J Virol. (2005) 79:10442–50. 10.1128/JVI.79.16.10442-10450.2005 16051836 PMC1182673

[234] Buie JJ Renaud LL Muise-Helmericks R Oates JC. IFN-alpha negatively regulates the expression of endothelial nitric oxide synthase and nitric oxide production: implications for systemic lupus erythematosus. J Immunol. (2017) 199:1979–88. 10.4049/jimmunol.1600108 28779021 PMC5587385

[235] Fedson DS, What treating Ebola means for pandemic influenza. J Public Health Policy. (2018) 39:268–82. 10.1057/s41271-018-0138-8 30013135 PMC7102163

[236] Fedson DS Opal SM Rordam OM. Hiding in plain sight: an approach to treating patients with severe COVID-19 infection. mBio. (2020) 11:1–3. 10.1128/mBio.00398-20 32198163 PMC7157814

